# C-terminal mini-PEGylation of a marine peptide N6 had potent antibacterial and anti-inflammatory properties against *Escherichia coli* and *Salmonella* strains in vitro and in vivo

**DOI:** 10.1186/s12866-022-02534-w

**Published:** 2022-05-12

**Authors:** Ting Li, Na Yang, Da Teng, Ruoyu Mao, Ya Hao, Xiumin Wang, Jianhua Wang

**Affiliations:** 1 Gene Engineering Laboratory, Feed Research Institute, Chinese Academy of Agricultural Sciences, Haidian District, 12 Zhongguancun Nandajie St, Beijing, 100081 People’s Republic of China; 2 Key Laboratory of Feed Biotechnology, Ministry of Agriculture and Rural Affairs, Beijing, 100081 People’s Republic of China

**Keywords:** Marine peptide-N6, PEGylation, Antibacterial activity, Anti-inflammatory activity, Intracorporeal distribution

## Abstract

**Background:**

Enteropathogenic *Escherichia coli* and *Salmonella pullorum* are two important groups of zoonotic pathogens. At present, the treatment of intestinal pathogenic bacteria infection mainly relies on antibiotics, which directly inhibit or kill the pathogenic bacteria. However, due to long-term irrational, excessive use or abuse, bacteria have developed different degrees of drug resistance. N6, an arenicin-3 derivative isolated from the lugworm, has potent antibacterial activity and is poorly resistant to enzymatic hydrolysis and distribution in vivo. Polyethylene glycol (PEG) is an extensively studied polymer and commonly used in protein or peptide drugs to improve their therapeutic potential. Here, we modified the N-/C-terminal or Cys residue of N6 with liner PEGn of different lengths (*n* = 2, 6,12, and 24), and the effects of PEGylation of N6 on the stability, toxicity, bactericidal mechanism, distribution and efficacy were investigated in vitro and in vivo.

**Results:**

The antimicrobial activity of the peptide showed that PEGylated N6 at the C-terminus (*n* = 2, N6-COOH-miniPEG) had potent activity against Gram-negative bacteria; PEGylated N6 at the N-terminus and Cys residues showed low or no activity with increasing lengths of PEG. N6-COOH-miniPEG has higher stability in trypsin than the parent peptide-N6. N6-COOH-miniPEG significantly regulated cytokine expression in lipopolysaccharides (LPS)-induced RAW 264.7 cells, and the levels of tumor necrosis factor-α (TNF-α), interleukin-6 (IL-6) and IL-1β were reduced by 31.21%, 65.62% and 44.12%, respectively, lower than those of N6 (-0.06%, -12.36% and -12.73%); N6-COOH-miniPEG increased the level of IL-10 (37.83%), higher than N6 (-10.21%). The data indicated that N6-COOH-miniPEG has more potent anti-inflammatory and immune-regulatory effect than N6 in LPS-stimulated RAW 264.7 cells. N6-COOH-miniPEG exhibited a much wider biodistribution in mice and prolonged in vivo half-time. FITC-labeled N6-COOH-miniPEG was distributed throughout the body of mice in the range of 0.75 – 2 h after injection, while FITC-labeled N6 only concentrated in the abdominal cavity of mice after injection, and the distribution range was narrow. N6-COOH-miniPEG improved the survival rates of mice challenged with *E. coli* or *S. pullorum*, downregulated the levels of TNF-α, IL-6, IL-1β and IL-10 in the serum of LPS-infected mice, and alleviated multiple-organ injuries (the liver, spleen, kidney, and lung), superior to antibiotics, but slightly inferior to N6.

**Conclusions:**

The antibacterial activity, bactericidal mechanism and cytotoxicity of N6-COOH-miniPEG and N6 were similar. N6-COOH-miniPEG has a higher resistance to trysin than N6. The distribution of N6-COOH-miniPEG in mice was superior to that of N6. In exploring the modulatory effects of antimicrobial peptides on cytokines, N6-COOH-miniPEG had stronger anti-inflammatory and immunomodulatory effects than N6. The results suggested that C-terminal PEGylated N6 may provide an opportunity for the development of effective anti-inflammatory and antibacterial peptides.

**Supplementary Information:**

The online version contains supplementary material available at 10.1186/s12866-022-02534-w.

## Background

Gram-negative pathogenic *Escherichia coli* and *Salmonella* are common pathogens of bacterial disease infections in livestock and poultry farming, which can cause diarrhea diseases and even sepsis in both animals and humans [1, 2]. Over four billion episodes of diarrhea occur annually in developing countries with diarrheagenic *E. coli* outbreaks also being reported [3]. *Salmonella* has been reported to cause between 200 million and 1.3 billion cases of intestinal disease worldwide each year [4], causing hundreds of millions of dollars in losses to the livestock and poultry industry each year [5]. Currently, the prevention and treatment of bacterial diarrheal diseases on farms mainly rely on antibiotics, but due to long-term unreasonable use, has led to varying degrees of bacterial resistance [6, 7]. Treatment with antibiotic therapy also induces the rapid release of high concentrations of LPS from the cell walls of gram-negative bacteria, and subsequently induces a variety of acute and chronic diseases [8]. Therefore, there is an urgent and growing need for the development of alternative antimicrobial agents with superior properties for the prevention and treatment of *E. coli* and *Salmonella* infections.

Antimicrobial peptides (AMPs) are promising therapeutic agents due to their rapid-killing and broad-spectrum antimicrobial properties [9]. AMPs may not cause widespread bacterial resistance due to their membranolytic mechanism that is distinct from conventional antibiotics, and thus they are a promising alternative to current antibiotics for disease treatment [10, 11]. However, some side effects of AMPs such as sensitivity to enzymes, unknown toxicity towards host cells, rapid renal clearance, potent immunogenicity and short circulation half-life have limited their application in clinics [12, 13]. Our preliminary study has demonstrated that N6 exhibited potent antimicrobial activity against gram-negative bacteria, especially *Salmonellae* and *E. coli* [14]. However, N6 showed poor trypsin resistance stability, and the inhibitory activity of N6 against *Salmonella* was reduced by 100% after 4 h of incubation with trypsin [14]. Therefore, improving the anti-trypsin capacity and circulating life of N6 is the main problem to solve the future clinical application of N6.

PEG, also known as a macrogols, is a polyether composed of repeated ethylene glycol units [-(CH_2_CH_2_O)_n_] with molecular weights (MWs) from 0.4 to 150 kDa; PEG has been used in pharmaceutical fields, consumer products, and cosmetics for decades [15]. PEGylation is a process through which PEG chains are conjugated to drug molecules to improve their in vivo efficacy and tissue distribution; PEGylation is one of the widely used technologies for proteins, peptides and small-molecule drugs due to its non-immunogenicity, degradability and excellent biocompatibility [16, 17]. PEG modification can provide more advantages in enhancing resistance to proteolytic degradation, prolonging blood circulation, improving water solubility, and enhancing pharmacokinetics of drugs [18, 19]. PEG molecules include a wide variety of linear, branched, Y-shaped, or multi-arm geometries forms; among them, linear PEGs are the simplest and most commonly used in bioconjugation and crosslinking of peptides or proteins [20]. Until now, a few AMPs have been covalently conjugated with PEG and they are currently under preclinical trials. Imura et al. [21] investigated the effects of PEGylation of tachyplesin I from *Tachypleus tridentatus* on the mechanism and found that the attachment of the large PEG moiety (5 kDa) did not change the mode of action of tachyplesin I, but its binding affinity to lipid bilayers was reduced by 2 – 3 fold. Morris et al. [22] synthesized PEG-CaLL at N-terminus, which is derived from LL-37 and cecropin A, and found that PEGylated CaLL derivatives had higher activity against *B. anthracis* (including vegetative and spore forms), *Staphylococcus aureus* and *Escherichia coli* than the parental LL-37 peptide. Benincasa et al. [23] modified Bac7 (1e35) by using cleavable ester bonds or non-hydrolyzable amide bonds and found that PEGylated Bac7 (1e35) improved the bioavailability and led to the wide distribution in mice and slow renal clearance of Bac7 (1e35). Singh et al. [24] found that PEG-binding to KYE28 effectively reduced the toxicity and serum protein clearance, increased selectivity, and retained anti-inflammatory effects. PEGylation at the C-terminus of the three-Lys-branching core of M33 led to an increase in stability to *Pseudomonas aeruginosa* elastase [25]. Meanwhile, other PEGylated AMPs such as M33 [25], KYE28 [24], CaLL [22], Nisin A [26], MA [27], tachyplesin I [21], magainin 2 [28], 73c [21] and Bac7(1–35) [23] improved solubility [29, 30], proteolytic stability [31, 32], and circulating half-life in the blood, respectively [33, 34]; they also reduced toxicity [35]. Noticeably, the conjugation site, distribution over the peptides and structure (linear, branched or dendritic) of PEG may influence antimicrobial effects and toxicity of drugs [24]. However, little is still known about how linear PEG length and localization affect the antibacterial and anti-inflammatory activity of AMPs in vitro and in vivo, respectively.

Considering this, in this study, a series of PEGylated N6 analogues were obtained by adding different lengths of linear PEGn (*n* = 2, 6, 12, and 24) with MWs from 145 to 1,127 Da to the N-terminus, C-terminus and Cys residues at positions 7 and 16 of a marine AMP-N6 (GFAWNVCVYRNGVRVCHRRAN), an AMP with potent bactericidal activity against Gram-negative bacteria in our previous study, but issues with protease sensitivity [14]. The effects of PEGn lengths (*n* = 2, 6, 12, and 24) at different sites on the bactericidal activity of N6 against major enteric pathogens-*E. coli* and *Salmonella pullorum* were firstly investigated, followed by their toxicity, stability, and mechanism of action. Furthermore, the in vivo therapeutic effect of PEGylated N6 was evaluated in ICR mice infected with *E. coli* and *S. pullorum*, respectively.

## Results

### Design of PEGylated N6 anlogues

N6 (2,477.8 Da) was modified by different lengths of linear PEGn (*n* = 2, 6, 12, and 24) with different MWs (145 – 1,127 Da) at the N-terminus, C-terminus or Cys residues at positions 7 and 16, generating N6-NH_2_-miniPEG (*n* = 2, MW of 2,620.93 Da), N6-NH_2_-PEG6 (*n* = 6, MW of 2,811.21 Da), N6-NH_2_-PEG12 (*n* = 12, MW of 3,075.54 Da), N6-NH_2_-PEG24 (*n* = 24, MW of 3,604.17 Da), N6-COOH-miniPEG (*n* = 2, MW of 2,620.93 Da), N6-Cys7-miniPEG (*n* = 2, MW of 2,620.93 Da), and N6-Cys16-miniPEG (*n* = 2, MW of 2,620.93 Da), respectively (Fig. S1, 2,3, 4,5,6,7,8 and Table 1). Among them, all PEGn-modification (*n* = 2, 6, 12, and 24) of N6 at the N- and C-terminus retained the primary structures of N6 (Fig. S2, 3,4,5 and 6). However, PEG-modification of N6 at Cys residues at positions 7 (N6-Cys7-miniPEG) and 16 (N6-Cys16-miniPEG) caused a significant change in structures (Fig. S7 and 8), which may further affect their antibacterial activity.

**Table 1:** Sequence and key properties of N6 and PEGylated N6 analogues

Peptides	Amino acid sequences (N’-C’)	Length (residues)	MW (Da)
N6	GFAWNVCVYRNGVRVCHRRAN	21	2477.80
N6-NH_2_-miniPEG	miniPEG-GFAWNVCVYRNGVRVCHRRAN	21	2620.93
N6-NH_2_-PEG6	PEG6-GFAWNVCVYRNGVRVCHRRAN	21	2811.21
N6-NH_2_-PEG12	PEG12-GFAWNVCVYRNGVRVCHRRAN	21	3075.54
N6-NH_2_-PEG24	PEG24-GFAWNVCVYRNGVRVCHRRAN	21	3604.17
N6-COOH-miniPEG	GFAWNVCVYRNGVRVCHRRAN-miniPEG	21	2620.93
N6-Cys7-miniPEG	GFAWNV-miniPEG-CVYRNGVRVCHRRAN	21	2620.93
N6-Cys16-miniPEG	GFAWNVCVYRNGVRVC-miniPEG-HRRAN	21	2620.93

miniPEG means HO(CH_2_CH_2_O)_2_H; PEG6 means HO(CH_2_CH_2_O)_6_H; PEG12 means HO(CH_2_CH_2_O)_12_H; PEG24 means HO(CH_2_CH_2_O)_24_H

### In vitro antibacterial activity

The antimicrobial activity of N6 and its PEGylated analogues against bacteria and *C. albicans* was determined by minimum inhibitory concentration (MIC) assay. As shown in Table 2, similar to N6, PEGylated N6 at the C-terminus (N6-COOH-miniPEG) showed higher antibacterial activity against gram-negative (with the MICs of 1.53 – 24.42 μM) than gram-positive bacteria (MICs >  = 24.42 μM). The MIC values of N6-COOH-miniPEG against *E. coli* and *Salmonella* were 1.53 – 3.05 and 3.05 – 6.1 μM, slightly higher than those of N6 (1.61 and 1.61 – 3.23 μM). A series of PEGylated N6 analogues at the N-terminus significantly reduced activity with increasing lengths of linear PEG (*n* = 2, 6, 12, and 24). The modification of N6 with Cys-linked PEG (N6-Cys7-miniPEG and N6-Cys16-miniPEG) lost antimicrobial activity (MIC > 48.84 μM), indicating that the disulfide bond in N6 may play a key role in antibacterial activity. Additionally, N6 and all PEGylated N6 analogues had lower or no activity against Gram-positive bacteria such as *S. aureus* and *S. hyicus* (MICs >  = 24.42 μM) and *C. albicans* CMCC98001 (MIC > 35.51 μM). The MIC results suggested that increasing the length of PEG at the N-terminus leads to a partial loss in antimicrobial activity of N6, but linear miniPEG (*n* = 2, with the size of 145 Da) modification at the C-terminus of N6 (N6-COOH-miniPEG) has potent antibacterial activity and can be used in the following experiments.

**Table 2:** MIC of the N6 and PEGylated N6 analogues

Species and strains	MIC															
	N6		N6-COOH-miniPEG		N6-NH_2_-miniPEG		N6-NH_2_-PEG6		N6-NH_2_-PEG12		N6-NH_2_-PEG24		N6-Cys7-miniPEG		N6-Cys16-miniPEG	
	μg/mL	μM	μg/mL	μM	μg/mL	μM	μg/mL	μM	μg/mL	μM	μg/mL	μM	μg/mL	μM	μg/mL	μM
Gram-negative bacteria																
* E. coli* CVCC195	4	1.61	8	3.05	16	6.1	16	5.69	32	10.4	128	35.51	> 128	> 48.84	128	48.84
* E. coli* CVCC1515	4	1.61	8	3.05	16	6.1	32	11.38	64	20.81	> 128	> 35.51	> 128	> 48.84	128	48.84
*E. coli* CVCC25922	4	1.61	4	1.53	8	3.05	8	2.85	16	5.2	128	35.51	> 128	> 48.84	128	48.84
* E. coli* ATCCO157	4	1.61	8	3.05	16	6.10	32	11.38	32	10.4	> 128	> 35.51	> 128	> 48.84	> 128	> 48.84
* S. typhimurium* ATCC14028	8	3.23	8	3.05	16	6.10	32	11.38	64	20.8	> 128	> 35.51	> 128	> 48.84	> 128	> 48.84
* S. pullorum* CVCC1802	4	1.61	8	3.05	16	6.10	16	5.69	32	10.4	128	35.51	> 128	> 48.84	128	48.84
* S. pullorum* CVCC1789	8	3.23	16	6.1	16	6.10	32	11.38	64	20.8	> 128	> 35.51	> 128	> 48.84	> 128	> 48.84
* S. enteritidis* CVCC3377	8	3.23	16	6.1	16	6.10	32	11.38	32	10.4	128	35.51	> 128	> 48.84	128	48.84
* S. pullorum* CVCC533	8	3.23	16	6.1	32	12.21	32	11.38	64	20.81	> 128	> 35.51	> 128	> 48.84	> 128	> 48.84
* P. aeruginosa* CICC21630	32	12.91	64	24.42	64	24.42	128	45.53	> 128	> 41.62	> 128	> 35.51	> 128	> 48.84	> 128	> 48.84
Gram-positive bacteria																
* S. aureus* ATCC43300	64	25.83	64	24.42	128	48.84	> 128	> 45.53	> 128	> 41.62	> 128	> 35.51	> 128	> 48.84	> 128	> 48.84
* S. aureus* ATCC546	64	25.83	128	48.84	128	48.84	> 128	> 45.53	> 128	> 41.62	> 128	> 35.51	> 128	> 48.84	> 128	> 48.84
* S. aureus* ATCC25923	64	25.83	128	48.84	> 128	> 48.84	> 128	> 45.53	> 128	> 41.62	> 128	> 35.51	> 128	> 48.84	> 128	> 48.84
* S. hyicus* NCTC10350	> 128	> 51.66	> 128	> 48.84	> 128	> 48.84	> 128	> 45.53	> 128	> 41.62	> 128	> 35.51	> 128	> 48.84	> 128	> 48.84
* S. hyicus* 437-2^a^	128	51.66	> 128	48.84	> 128	> 48.84	> 128	> 45.53	> 128	> 41.62	> 128	> 35.51	> 128	> 48.84	> 128	> 48.84
Fungus																
* C. albicans* CMCC98001	> 128	> 51.66	> 128	> 48.84	> 128	> 48.84	> 128	> 45.53	> 128	> 41.62	> 128	> 35.51	> 128	> 48.84	> 128	> 48.84

^a^Clinical isolated strain of Tianjin pig farm. *CVCC* China Veterinary Culture Collection Center, *ATCC* American Type Culture Collection, *CICC* China Center of Industrial Culture Collection, *NCTC* National Collection of Type Cultures, *CMCC* National Center for Medical Culture Collection

**Fig. 1:**
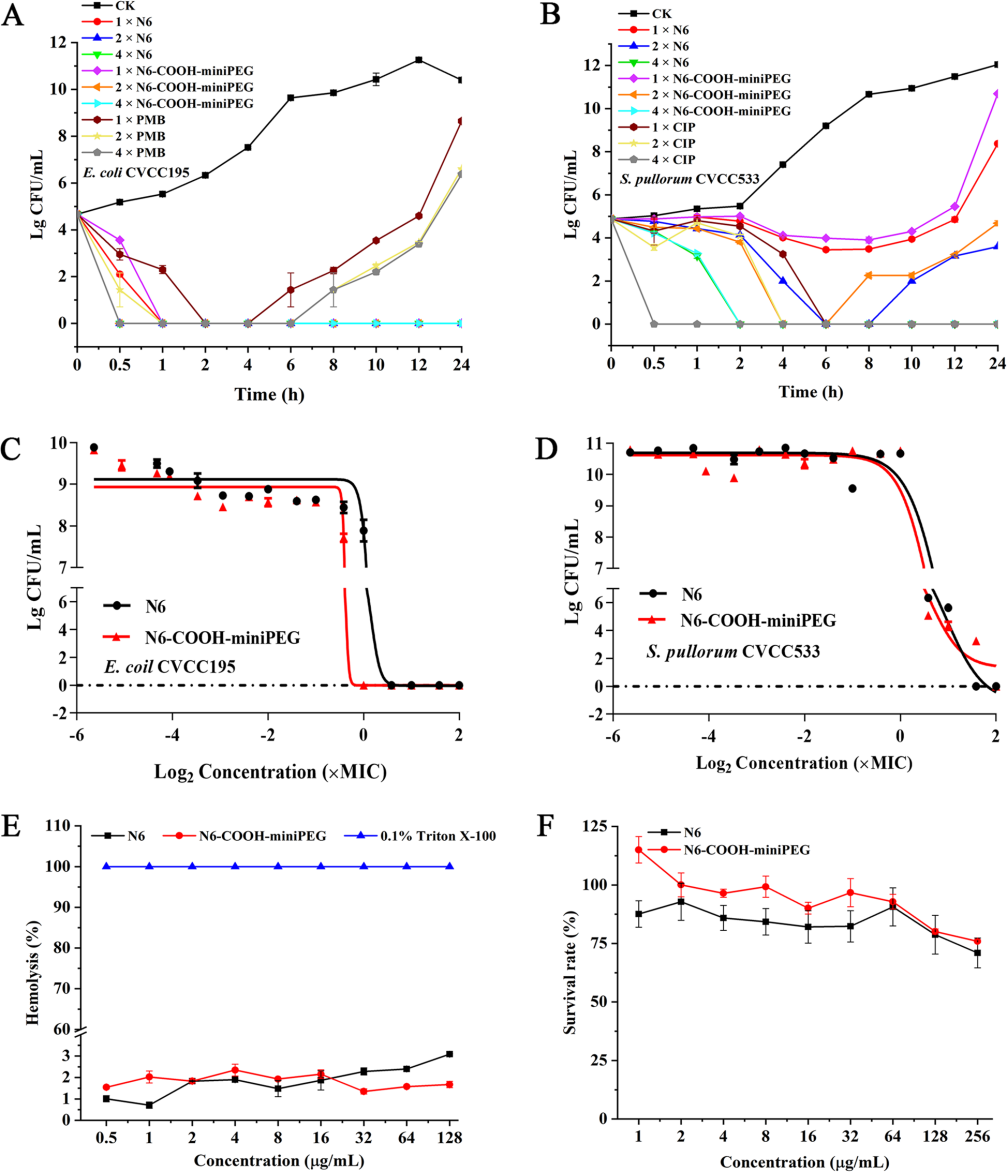
In vitro time/dose-dependent killing curves and toxicity of N6 and its N6-COOH-miniPEG. (**A**) Time-killing curves of peptides (1 × , 2 × , or 4 × MIC) against *E. coli* CVCC195. Negative control group (CK) is the bacteria solution with PBS instead of antimicrobial peptides. PMB was used as the positive control. (**B**) Time-killing curves of peptides (1 × , 2 × , or 4 × MIC) against *S. pullorum* CVCC533. PBS was used as the negative control. CIP was used as the positive control. (**C**) Dose-time curves of peptides against *E. coli* CVCC195. Results were given as mean ± SE (*n* = 3). (**D**) Dose-time curves of peptides against *S. pullorum* CVCC533. Results were given as mean ± SE (*n* = 3). (**E**) Hemolytic activity of peptides at different concentrations (0.5 – 128 μg/mL) against mouse erythrocytes. (**F**) Cytotoxicity of peptides toward RAW 264.7 cells

As shown in Fig. 1A, 1 × MIC N6 or N6-COOH-miniPEG killed 99.9% *E. coli* CVCC195 cells within 1 h (0.00 ± 0.00 Lg CFU/mL). Comparably, the *E. coli* CVCC195 treated with 1 × MIC PMB showed a slow reduction and started to regrow after 6 h (1.43 ± 0.72 Lg CFU/mL) (Additional File Sheet 1). The results showed that N6 with N6-COOH-miniPEG had stronger bactericidal activity than PMB. As shown in Fig. 1B, 2 × MIC N6 (4.14 ± 0.04 Lg CFU/mL) or N6-COOH-miniPEG (3.80 ± 0.08 Lg CFU/mL) did not completely inhibit *S. pullorum* CVCC533, 4 × MIC N6 and N6-COOH-miniPEG could kill 99.9% *S. pullorum* CVCC533 within 2 h (0.00 ± 0.00 Lg CFU/mL). Comparably, the bacteria treated with 1 × MIC CIP could kill 99.9% *S. pullorum* CVCC533 within 6 h (0.00 ± 0.00 Lg CFU/mL) (Additional File Sheet 2). Meanwhile, both N6 and N6-COOH-miniPEG showed significant concentration-dependent bactericidal activity against *E. coli* CVCC195 or *S. pullorum* CVCC533 (Fig. 1C and D).

**Fig. 2:**
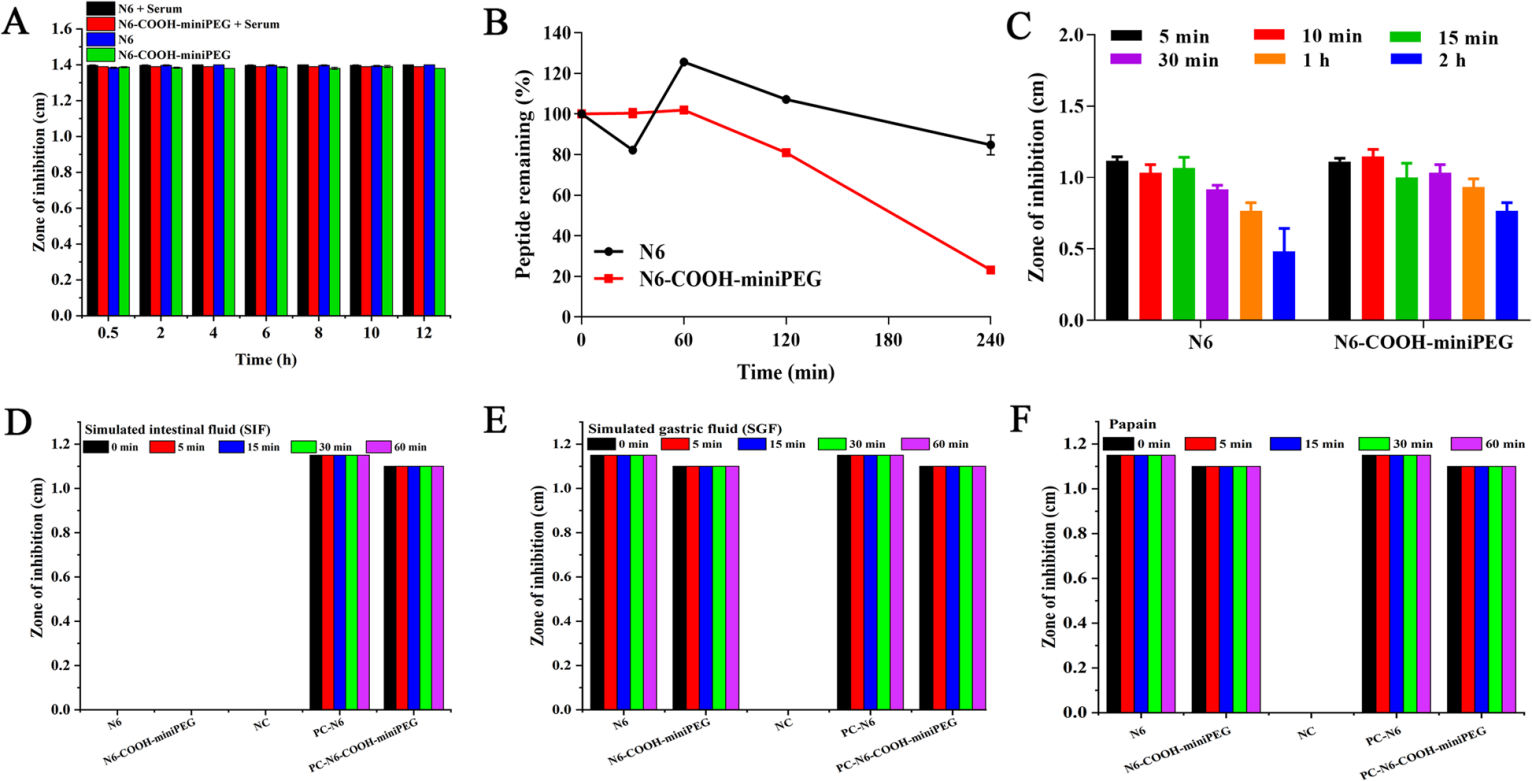
Stability of N6 and N6-COOH-miniPEG in different solutions. (**A**) Effects of serum on the antibacterial activity of N6 and N6-COOH-miniPEG against *E. coli* CVCC195. (**B**) The peptide remaining in serum. (**C**) The stability of peptides in trypsin buffer. (**D**) The stability of peptides in SIF. NC, SIF was used as a negative control. (**E**) The stability of peptides in SGF. NC, SGF was used as a negative control. (**F**) The stability of peptides in papain buffer. NC, papain was used as negative control. PC-N6, equivalent N6 prepared in PBS was used as positive control; PC-N6-COOH-miniPEG, equivalent N6-COOH-miniPEG prepared in PBS was used as positive control

### N6-COOH-miniPEG reduces cytotoxicity

As shown in Fig. 1E, the hemolysis rates of 0.5 – 128 μg/mL N6 and N6-COOH-miniPEG were 0.71 ± 0.15 - 3.08 ± 0.04% and 1.35 ± 0.12 - 2.35 ± 0.28%, respectively (Additional File Sheet 5), indicating that the effect of N6 and N6-COOH-miniPEG on hemolysis of mouse blood erythrocytes was extremely low. Meanwhile, at concentrations of 1 – 256 μg/mL, the cell survival rate of N6 was 70.93 ± 6.39 - 92.86 ± 7.92%, and the cell survival rate of N6-COOH-miniPEG is 75.95 ± 0.60 - 115.04 ± 5.69% (Additional File Sheet 6), overall, N6-COOH-miniPEG was slightly less cytotoxic than N6 for RAW 264.7 (Fig. 1F).

### N6-COOH-miniPEG improves stability toward trypsin and high temperatures

Stability of N6 and N6-COOH-miniPEG in different conditions (including temperature, pH value, enzyme, salt concentration, simulated gastric fluid (SGF) and simulated intestinal fluid (SIF)) was confirmed by the inhibition zone assay. As shown in Table S2, the antibacterial activity of N6 was slightly decreased after 1 h treatment at 80-100ºC, and the MIC values were increased by twofold (MIC_Control_ = 4 μg/mL, MIC_N6_ = 8 μg/mL), indicating that the antibacterial activity of N6 was reduced. However, the MIC values of N6-COOH-miniPEG were not affected after 1 h treatment at 80-100ºC (MIC_Control_ = 8 μg/mL, MIC_N6-COOH-miniPEG_ = 8 μg/mL), thus indicating that the thermal stability of N6-COOH-miniPEG was better than N6 at 80-100ºC. The antibacterial activity of N6 decreased at pH 4.0 (MIC_N6_ = 8 μg/mL) and 6.0 (MIC_N6_ = 8 μg/mL), while the antibacterial activity of N6-COOH-miniPEG only decreased at pH 4.0 (MIC_N6-COOH-miniPEG_ = 16 μg/mL) (Table S2). N6-COOH-miniPEG can retain its intrinsic activity against *E. coli* CVCC195 at different salt concentrations of 50–500 mM (MIC_N6-COOH-miniPEG_ = 8 μg/mL), while the MIC value of N6 is increased by two-fold (MIC_N6_ = 8 μg/mL), which proves that the salt ion stability of N6-COOH-miniPEG was better than N6 (Table S2).

Similar to N6, N6-COOH-miniPEG was resistant to pepsin (MIC = 8 μg/mL), but sensitive to proteinase K (MIC > 128 μg/mL) (Table S2). As shown in Fig. 2C, after treatment with trypsin for 2 h, N6 (0.48 ± 0.09 cm) and N6-COOH-miniPEG (0.77 ± 0.03 cm) retained 44.8% and 69.18% activity against *E. coli* CVCC195, respectively (Additional File Sheet 9), indicating that N6-COOH-miniPEG has better trypsin resistance stability than N6. After treatment with trypsin for 3 h, both N6 and N6-COOH-miniPEG lost antibacterial activity against *E. coli* CVCC195 (MIC > 128 μg/mL).

As shown in Fig. 2A and B, after treatment with 25% serum for 12 h, both N6 (1.40 ± 0.00 cm) and N6-COOH-miniPEG (1.39 ± 0.00 cm) retained their antimicrobial activity against *E. coli* CVCC195 (Additional File Sheet 7); however, the peptide content of N6-COOH-miniPEG (23.17 ± 0.14%) was lower than that of N6 (84.78 ± 2.83%) (Additional File Sheet 8). After a 1 h-incubation in simulated gastric fluid (SGF) or papain, both N6 (1.15 ± 0.00 cm) and N6-COOH-miniPEG (1.10 ± 0.00 cm) retained antimicrobial activity against *E. coli* CVCC195 (Additional File Sheets 11 and 12), which was immediately degraded in simulated intestinal fluid (SIF) (0.00 ± 0.00 cm) (Additional File Sheet 10) (Fig. 2D-F).

### Circular dichroism (CD) spectra of peptides

To analyze the structural features of N6 and N6-COOH-miniPEG, the CD spectra of peptides were measured in ddH_2_O, 20 mM sodium dodecyl sulfate (SDS), and 50% trifluoroethanol (TFE), respectively. The secondary structures of N6 and N6-COOH-miniPEG in ddH_2_O were characterized predominantly by random coils (37.9 – 41.2%), antiparallel strands (21.2 – 27.1%) and β-turns (26.5 – 29.2%) with a characteristic negative minimum at 205 nm and positive maximum at 190 nm and 230 nm, respectively (Fig. S9 and Table S1). The α-helix of N6-COOH-miniPEG (10.4%) in 20 mM SDS was higher than that of its parent peptide N6 (8.6%). The antiparallel of N6 or N6-COOH-miniPEG in 50% TFE was 32.9% and 42.6%, respectively (Additional File Sheet 25).

### N6-COOH-miniPEG potently permeabilize bacterial cell membranes and disrupt membrane potentials

The ability of peptides to permeabilize the outer membranes was measured with the N-phenyl-1-naphthylamine (NPN) fluorescent dye. As shown in Fig. S10A and B, both N6 and N6-COOH-miniPEG caused a time-dependent and concentration-dependent increase in fluorescence in *E. coli* CVCC195 and *S. pullorum* CVCC533 cells. As shown in Fig. S10A, both N6 and N6-COOH-miniPEG had disruptive effect on the outer membrane of *E. coli* CVCC195. The fluorescence intensity of 4 × MIC N6 (t = 1 min, fluorescence intensity = 17,884) and N6-COOH-miniPEG (t = 1 min, fluorescence intensity = 29,939) treated *E. coli* CVCC195 increased rapidly within 1 min, indicating that 4 × MIC N6 and N6-COOH-miniPEG could rapidly penetrate the outer membrane of *E. coli* CVCC195 within 1 min. As shown in Fig. S10B, 1 × MIC N6 (fluorescence intensity = 31,615) and N6-COOH-miniPEG (fluorescence intensity = 25,998) disrupted the outer membrane of *S. pullorum* CVCC533 faster, showing a strong fluorescence intensity at t = 0 s (Additional File Sheet 26).

The ability of peptides to permeabilize the inner membranes was measured with the propidium iodide (PI) fluorescence. As shown in Fig. S11A–B, the percentages of *E. coli* CVCC195 or S. *pullorum* CVCC533 stained with PI were 0.36–0.797% and 0.217 – 0.64% in the absence of N6 or N6-COOH-miniPEG, indicating intact bacterial inner membranes. After treatment with N6-COOH-miniPEG for 5–120 min, the percentages of PI-permeable *E. coli* CVCC195 cells were 4.89–14.9%, higher than those of N6 (3.03 – 15.5%), indicating a higher level of internalization of PEGylated N6. As shown in Fig. S11B, after treatment with 4 × MIC N6, the membrane permeabilizing ratio of bacteria increased to 26.1% (0.5 h) and 45.8% (2 h). After treatment with 4 × MIC N6-COOH-miniPEG for 0.5 – 2 h, the penetration ratio increased up to 29.2% and 35.9%, respectively. Therefore, the inner membrane disruption ability of N6-COOH-miniPEG is slightly lower than that of N6 against *S. pullorum* CVCC533.

To further examine effects of peptides on bacterial cell membranes, the membrane potentials were monitored with the voltage-sensitive anionic lipophilic dye 3,3′-dipropylthiadicarbocyanine iodide (DiSC_3_(5)) [36]. After treatment with 0.25–8 × MIC N6 and N6-COOH-miniPEG, the fluorescence values of *E. coli* CVCC195 and *S. pullorum* CVCC533 suspensions were enhanced rapidly and in a concentration-dependent manner (Fig. S12). This result demonstrated that N6 and N6-COOH-miniPEG could cause significant cell membrane depolarization in *E. coli* CVCC195 and *S. pullorum* CVCC533 cell membranes. Moreover, the fluorescence value after 8 × MIC N6-COOH-miniPEG (1168.67 ± 42.73 RFU) action was significantly higher than that of N6 (1037.67 ± 27.48 RFU) (Additional File Sheet 27).

### N6-COOH-miniPEG enhances the ability to bind to Lipopolysaccharide (LPS)

The ability of peptides to bind to LPS was determined using the fluorescent probe BODIPY-TR cadaverine (BC) displacement assay [28]. In the concentration range of 0.1 – 3 μM, both N6 and N6-COOH-miniPEG could displace the BC probe and bind to LPS in a dose-dependent manner (Fig. S13). N6-COOH-miniPEG has a better ability to bind LPS than N6 in the concentration range of 0.1 – 3 μM, except for 2 μM; at a concentration of 2 μM, the ability of N6-COOH-miniPEG to bind LPS with N6 was comparable (Additional File Sheet 28).

### N6-COOH-miniPEG more strongly affected the morphology and integrity of the bacteria

**Fig. 3:**
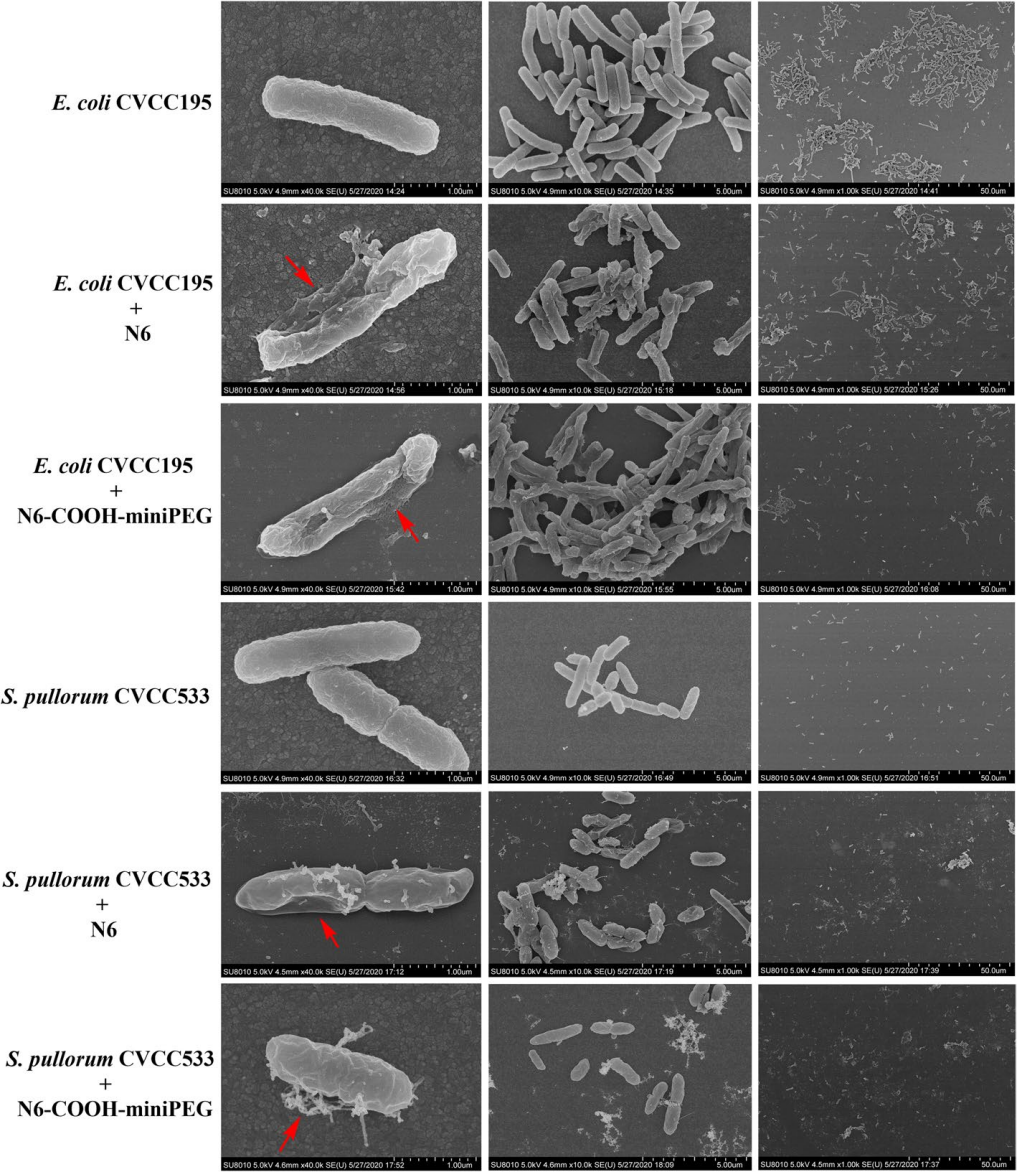
SEM images of *E. coli* CVCC195 and *S. pullorum* CVCC533 cells treated with N6 and N6-COOH-miniPEG. Bacteria in mid-logarithmic growth phases were treated with peptides at 4 × MIC for 2 h. After fixation and dehydration, the samples were observed on QUANTA200 SEM (FEI, Philips, Netherlands)

The morphology and integrity of *E. coli* CVCC195 and *S. pullorum* CVCC533 treated with the peptides were observed by scanning electron microscopy (SEM). In the untreated group, it was observed a normal cell morphology and smooth intact cell surface of *E. coli* and *S. pullorum* (Fig. 3). After treatment with 4 × MIC N6 and N6-COOH-miniPEG for 2 h, the cellular content leakage of bacterial cells was 55% and 63%, respectively. And cell shrinkage was also found in *E. coli* CVCC195. For *S. pullorum* CVCC533, after treatment with N6 and N6-COOH-miniPEG, the bacterial cells were deformed or collapsed, and filiferous substances were observed outside the cells. The percentages of *S. pullorum* CVCC533 showing leakage after the treatment of 4 × MIC N6 and 4 × MIC N6-COOH-miniPEG were 62% and 70%, repectively. After treatment with N6-COOH-miniPEG, the number of bacteria in the visual field was significantly lower than that in N6 treatment group (Red arrows: typical morphological changes such as crumpling or cytoplasmic leakage of the bacterium).

As shown in Fig. S14, in the absence of N6 and N6-COOH-miniPEG, smooth intact cell surfaces were observed in *E. coli* CVCC195 and *S. pullorum* CVCC533 cells. After exposure to N6 or N6-COOH-miniPEG for 2 h, the disruption of cell membranes and uneven electron cloud density and disruption of the internal structure were observed in both *E. coli* CVCC195 and *S. pullorum* CVCC533 cells. And the percentages of *E. coli* showing morphological changes after treatment with 4 × MIC N6 and 4 × MIC N6-COOH-miniPEG were 49% and 63%, respectively. The percentages of *S. pullorum* CVCC533 showing morphological changes after treatment with 4 × MIC N6 and 4 × MICN6-COOH-miniPEG were 61% and 65%, respectively.

### N6-COOH-miniPEG improves the anti-inflammatory properties in RAW 264.7 macrophages

**Fig. 4:**
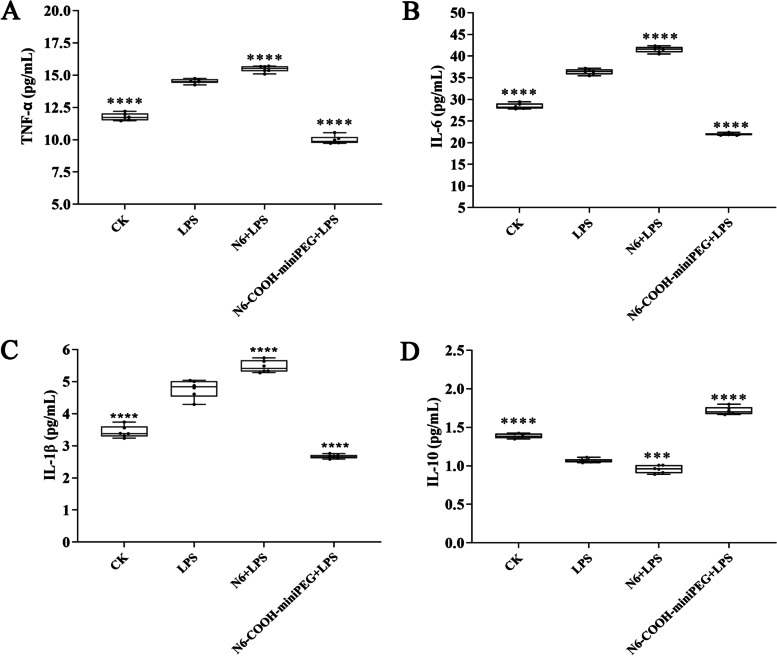
Effects of N6 and N6-COOH-miniPEG on inflammatory cytokines in RAW 264.7 cells. (**A**) TNF-α. (**B**) IL-6. (**C**) IL-1β. (**D**) IL-10. Cell were stimulated by LPS and treated by peptides. The concentrations of cytokines were measured by using an ELISA kit. The analyses were measured by one-way ANOVA, with Duncan's multiple comparisons test. A probability value of < 0.05 was considered significant. (*) Indicates the significance between control and treatment groups. *p < 0.05, **p < 0.01, ***p < 0.001, ****p < 0. 0001. (*) Indicates the significance between control and treatment groups; (**) Values represent as the mean ± SE of three independent experiments performed in triplicate

The effect of peptides on the expression of cytokines was investigated in LPS-induced RAW 264.7 cells by enzyme-linked immunosorbent assay (ELISA). As shown in Fig. 4, 100 ng/mL LPS treatment significantly induced the expression levels of TNF-α (18.9%), IL-6 (21.89%) and IL-1β (27.97%) in RAW 264.7 cells compared to the blank control. After treatment with 100 μg/mL N6-COOH-miniPEG, the levels of pro-inflammatory factors TNF-α, IL-6 and IL-1β were reduced by 31.21%, 65.62% and 44.12% compared to the LPS treatment group, respectively, which were superior to N6 (-0.06%, -12.36%, and -12.73%, respectively) (Additional File Sheets 13, 14, 15). In addition, after LPS stimulation, the level of anti-inflammatory cytokine IL-10 level (1.07 ± 0.01 pg/mL) in RAW 264.7 cells decreased by 23.09% compared to the blank control (1.39 ± 0.01 pg/mL); after treatment with N6-COOH-miniPEG, the IL-10 level (1.72 ± 0.02 pg/mL) increased by 37.83% compared to the LPS treatment group, higher than N6 (0.96 ± 0.02 pg/mL) (-10.21%) (Additional File Sheet 16). The data indicated that PEGylation of N6 at C-terminus has more potent anti-inflammatory and immune-regulatory effect than N6 in LPS-stimulated RAW 264.7 cells.

### N6-COOH-miniPEG has a much wider biodistribution in mice

**Fig. 5:**
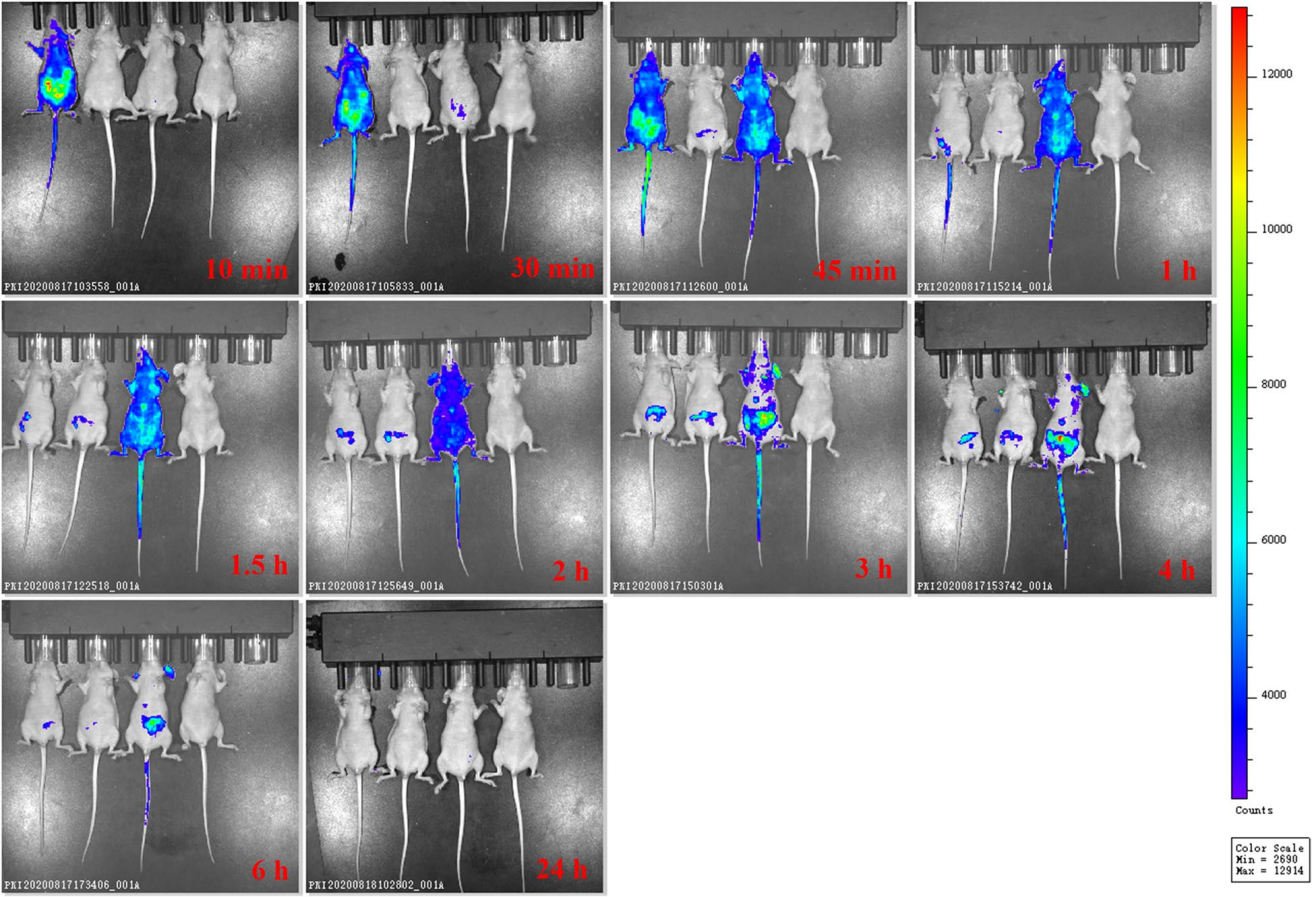
Biodistribution of N6 and N6-COOH-miniPEG in the healthy nude mice. The nude mice were injected intraperitoneally with 10 mg/kg FITC-labeled N6, N6-COOH-miniPEG or free FITC, and fluorescence (ventral) was observed at 10, 30, 45, 60, 90, 120, 180, 240, 360, and 1440 min, respectively. The mice from left to right were free FITC, FITC-labeled N6, FITC-labeled N6-COOH-miniPEG, and the blank control, respectively

As shown in Fig. 5, free fluorescein isothiocyanate (FITC) in the control mice was rapidly distributed within 10 min and showed a slower renal clearance at 6 h post injection. The weak fluorescence of FITC-N6 was accumulated in the abdomen of mice after injection and the fluorescence did not distribute throughout the body. Comparably, however, strong fluorescence of FITC-N6-COOH-miniPEG was distributed throughout the body at 45 min post injection; it decreased after 3 h and disappeared after 24 h, indicating a more extensive distribution of N6 after PEGylation at the C-terminus and a slower renal clearance compared to the unmodified peptide. Moreover, it was observed a significant concentration of the PEGylated N6 in kidney even at 24 h after injection (Fig. 5), indicating a prolonged in vivo half-time for N6 after its conjugation to miniPEG.

### N6-COOH-miniPEG protects mice from lethal challenge with *E. coli *CVCC195 or *S. pullorum* CVCC533

**Fig. 6:**
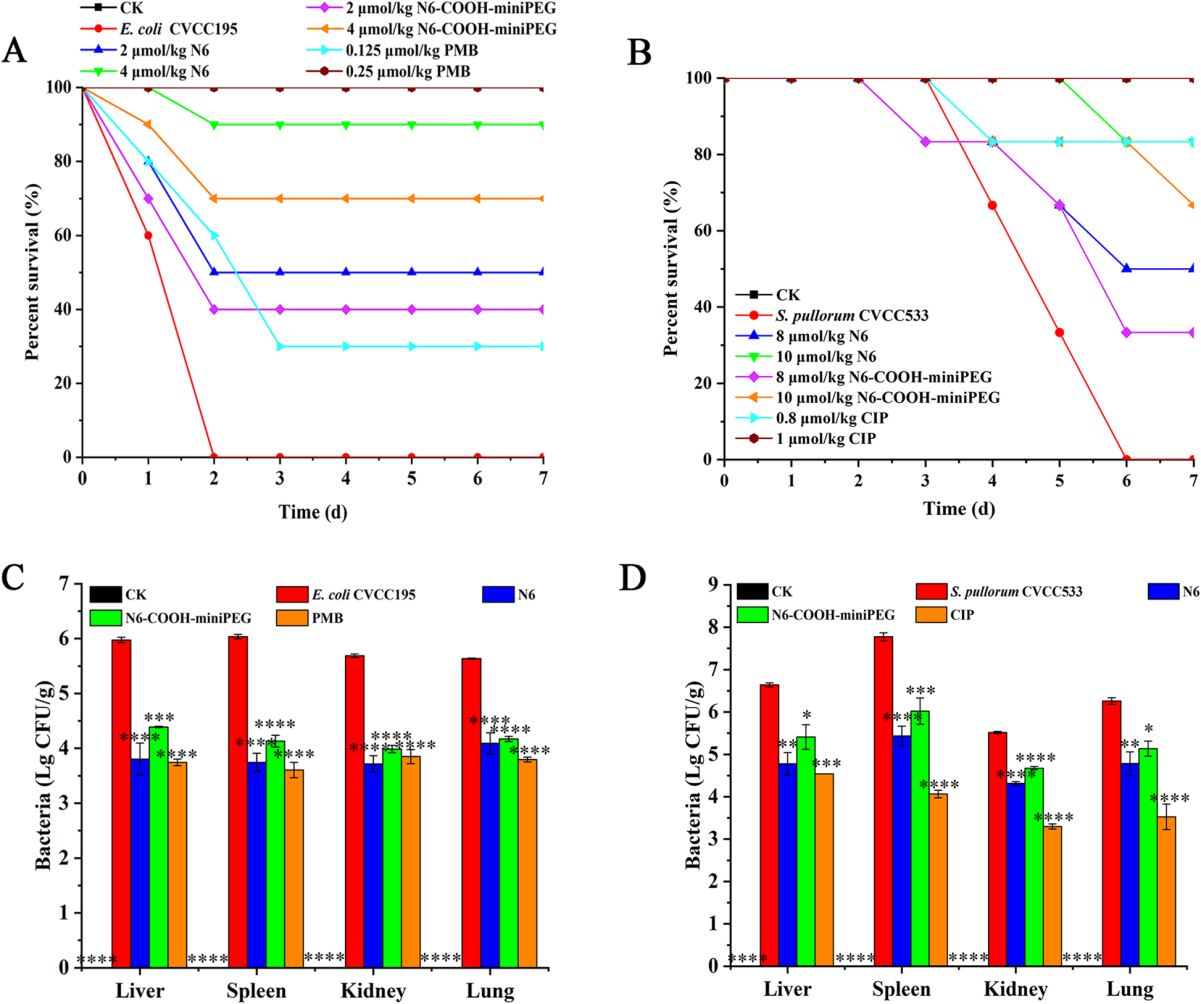
N6/N6-COOH-miniPEG protected mice from lethal challenge with *E. coli* and *S. pullorum*. Survival of mice treated with N6 and N6-COOH-miniPEG in (**A**) *E. coli* and (**B**) *S. pullorum* lethal models. Mice were intraperitoneally injected with *E. coli* (1 × 10^9^ CFU/mL) followed by N6, N6-COOH-miniPEG or PMB at 0.5 and 8 h; Mice were intraperitoneally injected with *S. pullorum* (5 × 10^7^ CFU/mL) followed by N6, N6-COOH-miniPEG or CIP at 0.5 and 8 h. Survival rates were recorded for 7 days. Effects on the bacterial translocation. Bacteria were counted in the livers, spleens, kidneys and lungs of mice infected with (**C**) *E. coli* or (**D**) *S. pullorum* after treatment with N6 or N6-COOH-miniPEG. The untreated mice were used as the negative control. Data were expressed as mean ± SE (n = 3)

To evaluate efficacy of N6 and N6-COOH-miniPEG in a peritonitis model, the mice were inoculated intraperitoneally with *E. coli* CVCC195 and *S. pullorum* CVCC533, respectively and treated with peptides. The uninoculated mice of the control group survived throughout the experimental period (Fig. 6A-B). As shown in Fig. 6A, all mice infected with *E. coli* CVCC195 in the control group died within 2 d. After treatment with 2 and 4 μmol/kg N6, the survival rates of mice were 50% and 90%, respectively, higher than those of N6-COOH-miniPEG (40% and 70%) and 0.125 μmol/kg polymyxin B (PMB) (30%). The survival rate of mice treated with 0.25 μmol/kg PMB was 100% (Additional File Sheet 17). The mice infected with *S. pullorum* CVCC533 in the control group developed the symptoms of diarrhea and weight loss, and all died within 6 d. After treatment with 8 and 10 μmol/kg N6, the survival rates of mice were 50% and 83.33%, respectively, higher than those of N6-COOH-miniPEG (33.33% and 66.67%) (Additional File Sheet 18). The survival rate of mice treated with 1 μmol/kg CIP was 100% (Fig. 6B). The results implied that PEGylation of N6 at the C-terminus slightly reduced the efficacy of the N6 in vivo.

To investigate the effects of peptides on translocation of *E. coli* CVCC195 or *S. pullorum* CVCC533 from the peritoneal cavity to other organs, livers, spleens, kidneys and lungs were collected and analyzed by the plate count method. As shown in Fig. 6C, in the untreated mice infected with *E. coli* CVCC195, the bacterial counts in the livers, spleens, kidneys and lungs were 5.97 ± 0.05, 6.04 ± 0.04, 5.69 ± 0.03, and 5.64 ± 0.01 Lg CFU/g, respectively (Additional File Sheet 19). After treatment with 4 μmol/kg N6-COOH-miniPEG, *E. coli* CVCC195 cells in livers, spleens, kidneys and lungs were significant decreased by 26.53%, 31.56%, 29.91% and 25.95%, respectively, which was lower than those of 4 μmol/kg N6 (36.30%, 38.00%, 34.63%, and 27.4%) and 0.25 μmol/kg PMB (37.35%, 40.3%, 32.29%, and 32.64%). The untreated mice infected with *S. pullorum* CVCC533, the bacterial counts in the livers, spleens, kidneys and lungs were 6.64 ± 0.05, 7.78 ± 0.09, 5.52 ± 0.03, and 6.26 ± 0.08 Lg CFU/g, respectively (Additional File Sheet 20). After treatment with 10 μmol/kg N6-COOH-miniPEG, *S. pullorum* CVCC533 cells in the livers, spleens, kidneys and lungs were significantly decreased by 18.52%, 22.58%, 15.27%, and 17.95%, respectively, lower than those of N6 (28.00%, 30.21%, 2179%, and 23.58%) and 1 μmol/kg CIP (31.66%, 47.75%, 40.20%, and 43.65%) (Fig. 6D). The results suggested that N6-COOH-miniPEG is less able to inhibit bacterial translocation in mice than N6 and antibiotics.

**Fig. 7:**
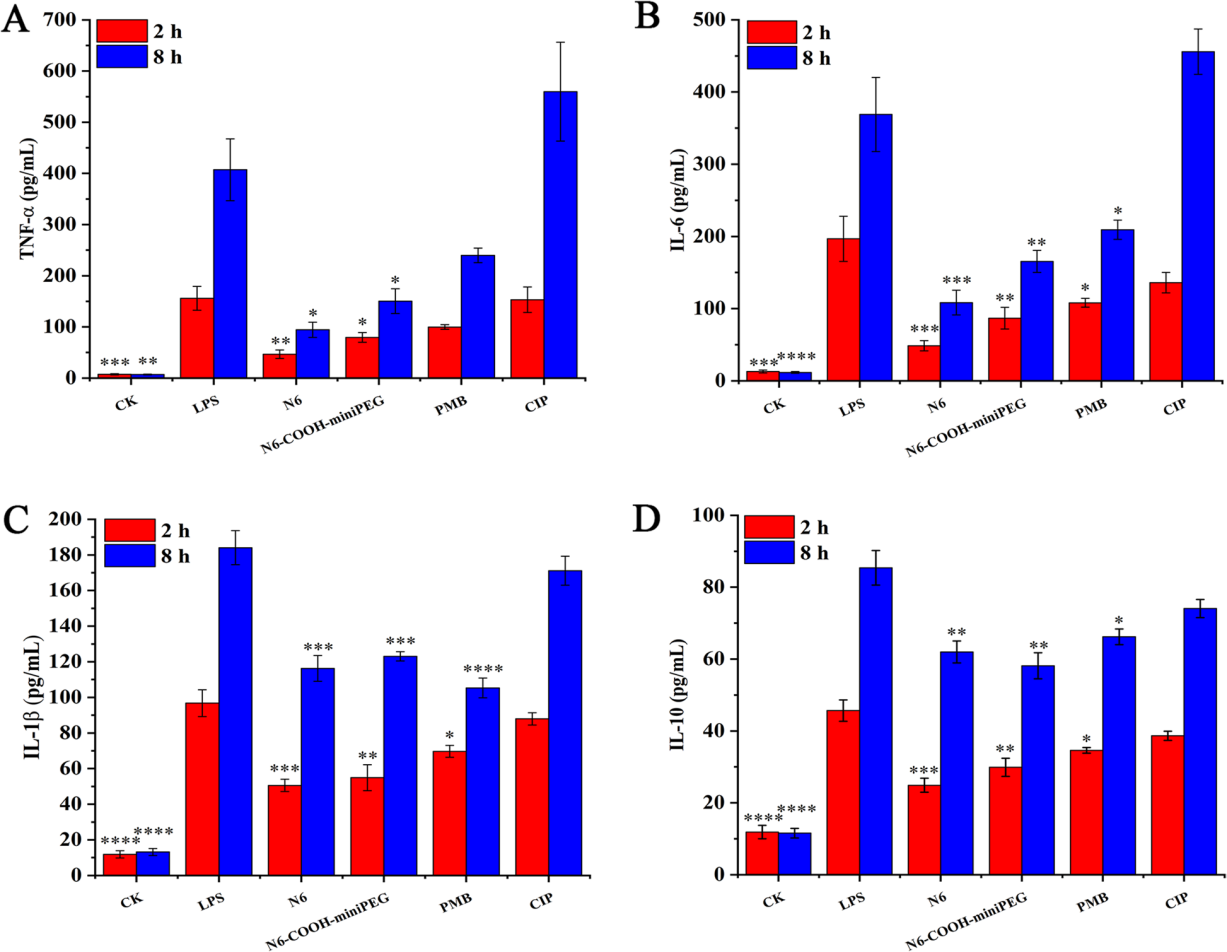
Effects on cytokines in mice. Mice were challenged with LPS (10 mg/kg, 200 μL), followed by injection with N6 or N6-COOH-miniPEG (0.25 μmol/kg). Sera were collected and the levels of (**A**) TNF-α, (**B**) IL-6, (**C**) IL-1β, and (**D**) IL-10 were detected by using an ELISA kit after 2 and 8 h after treatment. The analyses were measured by one-way ANOVA, with Duncan’s multiple comparisons test. A probability value of < 0.05 was considered significant. (*) Indicates the significance between control and treatment groups. *p < 0.05, **p < 0.01, ***p < 0.001, ****p < 0. 0001. (*) Indicates the significance between control and treatment groups; (**)

To further explore the effects of N6 and N6-COOH-miniPEG on inflammatory cytokines, an endotoxemia mouse model was constructed by intraperitoneal injection with 10 mg/kg LPS, followed by treatment with peptides or antibiotics. As shown in Fig. 7A-D, a significant increase of TNF-α (155.77 ± 23.38 – 407.17 ± 60.36 pg/mL), IL-6 (196.67 ± 31.24 – 368.87 ± 51.44 pg/mL), IL-1β (96.77 ± 7.52 –184.07 ± 9.60 pg/mL) and IL-10 (45.67 ± 2.99 – 85.40 ± 4.83 pg/mL) was observed in the LPS-infected mice, as compared to the blank control group (7.05 ± 0.72 – 7.40 ± 1.23, 11.63 ± 1.19 – 12.97 ± 2.03, 11.87 ± 2.00 – 13.17 ± 1.96, and 11.57 ± 1.33 – 11.87 ± 1.89 pg/mL) (Additional File Sheets 21, 22, 23, 24). After 2 h of treatment with N6-COOH-miniPEG, the TNF-α, IL-6, IL-1β and IL-10 levels decreased by 49.03%, 55.85%, 43.23%, and 34.53%, respectively, lower than those of N6 (70.15%, 75.34%, 47.74%, and 45.47%), but higher than those of PMB (35.89%, 45.05%, 27.97%, and 24.23%) and CIP (1.78%, 30.83%, 9.13%, and 15.33%). After 8 h of treatment with N6-COOH-miniPEG, the TNF-α, IL-6, IL-1β and IL-10 levels reduced by 66.11%, 55.17%, 33.14%, and 31.93%, respectively, lower than those of N6 (76.79%, 70.64%, 36.82%, and 27.44%), but higher than those of PMB (41.11%, 43.27%, 42.79%, and 22.48%) and CIP (-0.37%, -0.24%, 0.07%, and 0.13%) (Additional File Sheets 21, 22, 23, 24). These results suggested that both N6 and N6-COOH-miniPEG can regulate LPS-induced cytokines, and both are far superior to antibiotics.

**Fig. 8:**
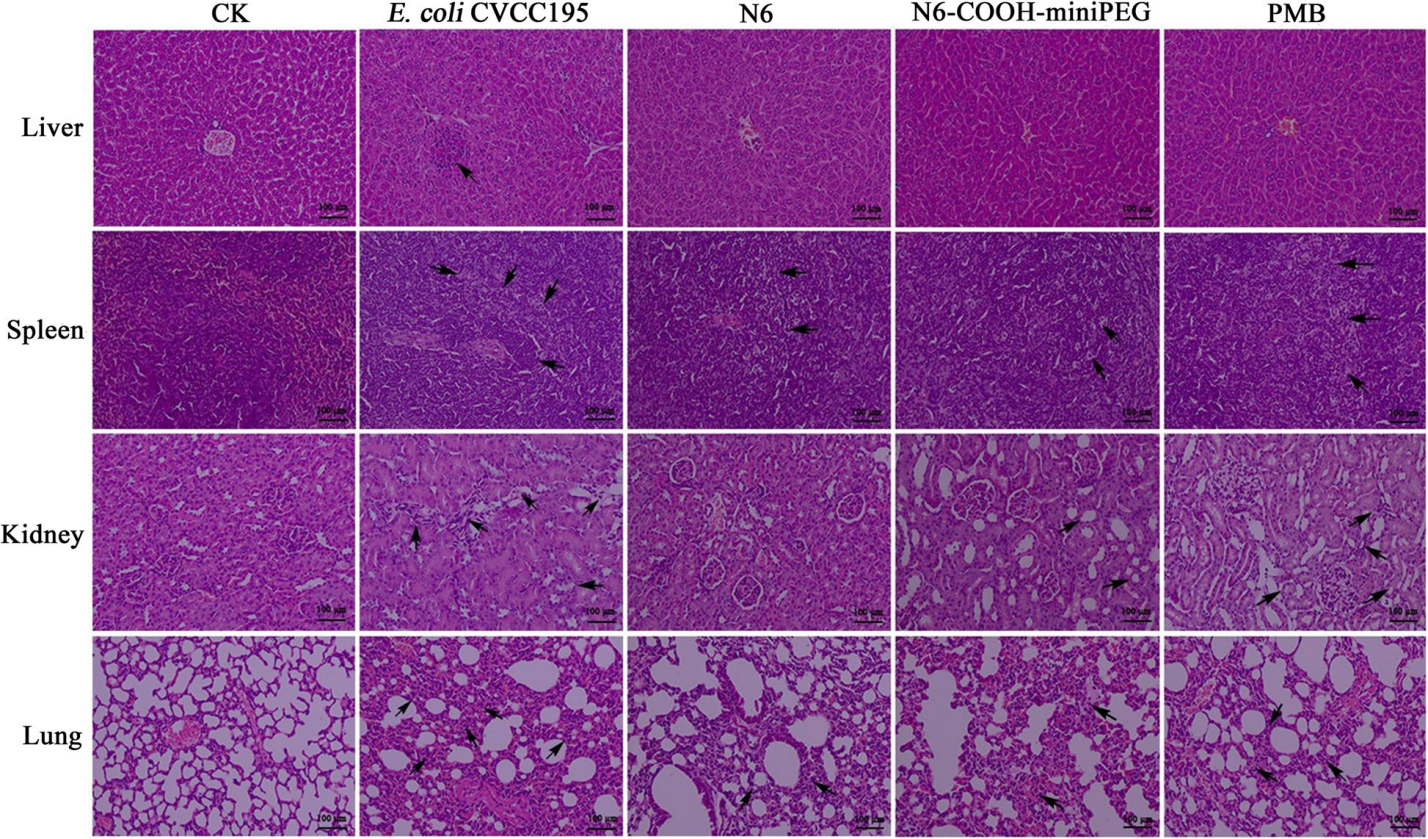
Effects of N6 and N6-COOH-miniPEG on organ injury in mice. Mice were infected intraperitoneally with *E. coli* CVCC195 (1 × 10^9^ CFU/mL, 200 μL) and treated with N6 (4 μmol/kg) or N6-COOH-miniPEG (4 μmol/kg). Livers, spleens, kidneys and lungs were harvested from sacrificed mice at 24 h after infection. CK group: The livers, spleens, kidneys, and lungs were normal; Infected *E. coli* CVCC195 group: Inflammatory lesions such as foci of hepatocellular necrosis, reactive enlargement of small splenic nodules, localized foci of interstitial inflammation in the kidney, and diffuse inflammatory lesions in the interstitial lung (arrow) (× 200, scale bar = 100 μm); N6 treatment group: There is a reduced density of lymphocytes in the local splenic nodules and a small infiltration of inflammatory cells in the interstitial lung (arrow) (× 200, scale bar = 100 μm); N6-COOH-miniPEG treatment group: The splenic nodules are mildly enlarged, the renal tubules are partially atrophied and a small amount of inflammatory cell infiltration is present in the interstitial lung (arrow) (× 200, scale bar = 100 μm); PMB treatment group: Reactive enlargement of splenic nodules, atrophy of the renal tubules at the site of the lesion and infiltration of inflammatory cells in the interstitial lung (arrow) (× 200, scale bar = 100 μm)

To investigate whether N6 and N6-COOH-miniPEG alleviate organ injury in mice challenged with *E. coli* CVCC195 and *S. pullorum* CVCC533, the livers, spleens, kidneys and lungs were dissected from mice at different times after treatment with peptides or antibiotics. As shown in Fig. 8 and Fig. S15, in the uninfected group, no pathological symptoms were observed in the organs of the mice. However, the untreated mice infected with *E. coli* CVCC195 developed severe organ damage, especially in the lungs where tissue destruction was evident, with massive inflammatory exudate in the alveolar cavity. In contrast, after treatment with N6, N6-COOH-miniPEG and PMB, there was apparently less damage of liver, spleen, kidney and lung at 24 h and 5 d post-treatment. As shown in Fig. S16 and 17, the untreated mice infected with *S. pullorum* CVCC533 developed acute injury, characterized by a large number of inflammatory cells, degeneration, cord atrophy or necrosis in the tissues. In contrast, after treatment with N6, N6-COOH-miniPEG and CIP, there was apparently less damage of liver, spleen, kidney and lung at 48 h and 4 d post-treatment. The efficacy of N6-COOH-miniPEG was slightly lower than that of N6 and antibiotics. The results suggested that N6-COOH-miniPEG improves the histopathology of mouse tissue damage induced by *E. coli* or *S. pullorum*, superior to antibiotics, but inferior to N6.

## Discussion

AMPs are a class of small cationic polypeptides that are important components of biological innate immunity [37]; AMPs are thought to be effectively conserved immune molecules in ancient evolutionary mammals [38, 39] and thus have become the most promising new generation of drugs to replace traditional antibiotics due to their broad antibacterial spectrum [37], high antibacterial activity [40, 41], and low resistance [42, 43]. To date, however, few AMPs has been approved by the US Food and Drug Administration (FDA) due to their toxicity, protease cleavage, and short half-life [11, 34, 43, 44]. Our preliminary study has demonstrated that N6 exhibits low toxicity to eukaryotic cells, low hemolysis, and potent antimicrobial activity against Gram-negative bacteria, especially for *Salmonellae* and *E. coli* [14], however, N6 is completely degraded by trypsin [41]. Therefore, in this study, PEGylated N6 was generated by adding the different lengths of linear PEGn (n = 2, 6, 12, and 24) to N-/C-terminus or Cys residue, and their antibacterial/anti-inflammatory activity, stability, mechanism and efficacy were systematically evaluated in vitro and in vivo.

PEGylation defines the modification of protein, peptide or non-peptide molecules by the linking of one or more PEG molecules [19]. The PEG polymer is non-toxic, non-immunogenic, non-antigenic and highly soluble in water and has been approved by FDA to modify many drugs [45]. In this study, we firstly attached linear PEGn (*n* = 2, 6, 12, and 24) with different MWs of 145- to 1,127-Da to N-/C- terminus and Cys residues at positions 7 and 16 of a marine peptide N6 to improve stability. The antibacterial activity of PEGylated N6 showed that N6 modified by linear miniPEG (*n* = 2, MW of 145 Da) at the C-terminus (N6-COOH-miniPEG) had the highest antibacterial activity against Gram-negative bacteria (MICs of 1.53 – 6.1 μM) (Table 2), which may be related to PEGylation of peptides [46–48]. Conversely, all PEGylation of N6 at the N-terminus had low/no antimicrobial activity (Table 2), which is agreement with the previous study in which N-terminal modification of glucose-dependent insulinotropic polypeptide (GIP) (1–30) with 40 kDa PEG lost functional activity; C-terminal PEGylation of GIP (1–30) retained agonism and reasonable potency at the GIP receptor and conferred a high level of DPPIV resistance [49]. Additionally, antibacterial activity of PEGylated N6 reduced with the increase in PEG length (MW of 145 – 1,127 Da) at the N-terminus (Table 2), which was agreement with PEGylated KYE28 [24]. Both N6-Cys7-miniPEG and N6-Cys16-miniPEG with the PEG modification at Cys residues (Cys7 and Cys16) lost the antibacterial properties against gram-negative and gram-positive bacteria (with MICs > 128 μg/mL) (Table 2), indicating that it may be related to PEGylation that change structural characteristics of peptides at the specific site (Fig. S7 and 8). Some previous studies showed that PEGylation of AMPs such as CaLL and aurein 2.2 could inhibit α-helix formation [22, 50]. In this study, PEGylation of N6 at the C-terminus (N6-COOH-miniPEG) did not have a detrimental impact upon α-helical content in ddH_2_O (with a decrease of 0.1%), 20 mM SDS (with an increase of 1.8%), and 50% TFE (with an increase of 2.2%); however, the antiparallel of N6-COOH-miniPEG was increased by 5.9% (ddH_2_O), 13% (SDS), and 10.3% (TFE), respectively when compared to N6 (Table S1). The result indicated that PEGylation of N6 may alter the secondary structure of the peptide and thereby it may affect antibacterial activity.

The mechanisms of action of AMPs against bacteria include both membrane-breaking and non-membrane breaking structures [51]. In a previous study, Yang et al. [14] have demonstrated that marine peptide N6 has a significant disruptive effect on the inner and outer membranes of *E. coli* CVCC195. In this study, by further investigating the effect of miniPEG modification on the bactericidal mechanism of N6, it was found that N6-COOH-miniPEG disrupted the internal and external membrane of bacteria to similar extent as N6 (Fig. S10 and 11). The result was also observed in SEM and transmission electron microscopy (TEM) (Fig. 3 and S14), respectively. However, we were surprised to find that high concentrations of N6-COOH-miniPEG had a stronger effect on bacterial inner membrane permeability (Fig. S11) and inner membrane depolarization (Fig. S12) than N6. It may be related to miniPEG attachment, which was more favorable for N6 to shift to a α-helical conformation, which in turn may disrupt the bacterial cell membrane and cause bacterial death (Fig. S9 and Table S1) [14, 24, 28]. Meanwhile, miniPEG linkage may stabilize the ordered conformation of N6, which was consistent with the findings of previous reports [22]. Additionally, N6-COOH-miniPEG more strongly bound to bacterial LPS than N6 (Fig. S13) and thereby it may prevent inflammatory responses.

PEGylation via amine modification is known to be a reliable method for effectively shielding proteolysis of peptides [52, 53]. Theoretically, there should be a difference in the amount of degradation between the native and mini-PEGylated peptide because of PEG interference of the trypsin cleavage process [54]. In our study, we found that miniPEG conjugation prolonged the activity of N6 in trypsin buffer and improved the thermal stability of N6 (Fig. 2C and Table S2). This result was consistent with the finding that PEGylation improved the anti-trypsin stability of the recombinant interferon (rhIFN) α-2a [55]. Furthermore, this result was consistent with the finding that PEGylation improved the thermal stability of the model protein CT-322 and PEGylated CT-322 could refold correctly after thermal denaturation [56].

The in vivo distribution of peptides showed that mini-PEGylation of N6 had a wider distribution and a slower renal clearance than unmodified N6 (Fig. 5), indicating a prolonged in vivo half-life of N6 after binding to linear PEG [34, 57]. This is mainly because PEGylation offers the possibility to specifically protect endangered termini and furthermore increases molecular mass, leading to slow absorption after injection, reduced glomerular filtration and lower renal excretion, thus indirectly increasing the in vivo half-life of the short peptide [58, 59]. Furthermore, it has already been demonstrated that the effect of protecting PEGylated proteins from proteolysis is especially strong when high-molecular-weight, branched PEGs are used [60]. Moreover, N6-COOH-miniPEG was found to have a stronger anti-inflammatory function than N6 in RAW 264.7 cells (Fig. 4). However, N6-COOH-miniPEG showed a slightly lower efficacy in mice infected with *E. coli* CVCC195 or *S. pullorum* CVCC533 than N6; this result was consistent with the finding that PEGylated peptides such as tachyplesin I substantially inhibited antibacterial effects against *S. epidermidis*, *E. coli* and other strains [21]. The reduced antimicrobial activity of PEGylation of peptides may be due to a decrease in the number of protonated amino groups and shielding of the positive charges by PEG chains, leading to decreasing in electrostatic interactions of peptides with the negatively charged bacterial surface [61].

## Conclusions

In this study, we designed PEGylated peptides based on a marine peptide-N6 and examined the effects of different lengths of PEGn (*n* = 2, 6, 12, and 24) modification at different sites on the toxicity, stability, mechanism, distribution, and in vitro and in vivo antibacterial/anti-inflammatory activities of N6. The results showed that PEGylated N6 at the C-terminus (*n* = 2, N6-COOH-miniPEG) significantly improved proteolytic stability. N6-COOH-miniPEG more potently bound to LPS and prolonged in vivo retention than N6; N6-COOH-miniPEG retained the anti-inflammatory activity of the parent peptide N6. N6-COOH-miniPEG showed a slightly lower efficacy in mice than N6. The results suggested that PEGylation of N6 contributes to the design of effective anti-inflammatory and antibacterial PEGylated peptides for clinical applications.

## Methods

### Strains, peptides, and animals

The strains of *E. coli* CVCC195, *E. coli* CVCC1515, *E. coli* CVCC25922, *E. coli* ATCCO157, *S. enteritidis* CVCC3377, *S. pullorum* CVCC533, *S. pullorum* CVCC1789 and *S. pullorum* CVCC1802 were purchased from China Veterinary Culture Collection Center (CVCC). *S. typhimurium* ATCC14028, *S. aureus* ATCC546 and *S. aureus* ATCC25923 were purchased from American Type Culture Collection (ATCC). *P. aeruginosa* CICC21630 were purchased from China center of industrial culture collection (CICC). *Candida albicans* CMCC98001 was purchased from National Center for Medical Culture Collections. One clinical strain of *S. hyicus* 437–2 was obtained from Animal Husbandry and Veterinary Research Institute (Tianjin, China). N6 and its PEGylated analogues with different lengths of PEGn (*n* = 2, 6, 12, and 24) were synthesized by Mimotopes (Wuxi, China) and WuXi App Tec (Shanghai, China), respectively. The purity of all peptides was greater than 90%. Six-week-old specific-pathogen-free (SPF) female ICR and nude mice (approximately 20 ± 2 g/mouse) were obtained from the Vital River Laboratories (VRL, Beijing, China). All other chemical reagents were of analytical grade.

### Design of PEGylated N6 analogues

A marine peptide-N6 was attached by different lengths of linear PEGn (*n* = 2, 6, 12, and 24) with MWs from 145 to 1,127 Da to the N-terminus, C-terminus and Cys residues at position of 7 and 16; they are named as N6-NH_2_-miniPEG (*n* = 2, at the N-terminus), N6-NH_2_-PEG6 (*n* = 6, at the N-terminus), N6-NH_2_-PEG12 (*n* = 12, at the N-terminus), N6-NH_2_-PEG24 (*n* = 24, at the N-terminus), N6-COOH-miniPEG (*n* = 2, at the C-terminus), N6-Cys7-miniPEG (*n* = 2, at residue Cys7), and N6-Cys16-miniPEG (*n* = 2, at residue Cys16), respectively (Table 1). After synthesis and purification, the MWs of N6 and PEGylated N6 analogues were measured by electrospray ionization mass spectrometry (ESI–MS).

### Antibacterial activities and bactericidal curves of N6 and PEGylated N6 analogues

The MIC was determined according to the method developed by Clinical and Laboratory Standards Institute (CLSI) [62]. The strains were cultured in Mueller–Hinton Broth medium (MHB) at the appropriate temperature until mid-log phase. A series of two-fold dilutions of N6 and PEGylated N6 (10 μL) and bacterial suspension (1 × 10^5^ CFU/mL, 90 μL) were added into 96-well plates and incubated at 37 °C for 18 – 24 h. PBS and MHB were used as the negative and blank control, respectively. All assays were conducted in triplicate.

The *E. coli* CVCC195 and *S. pullorum* CVCC533 cells were cultivated to mid-log phase at 37 °C (250 rpm) (1 × 10^5^ CFU/mL) and incubated with 1 × , 2 × , or 4 × MIC N6 and N6-COOH-miniPEG. The sample (100 μL) was taken from each flask at 0, 0.5, 1, 2, 4, 6, 8, 10, 12, and 24 h, respectively and serially diluted for colony counting [63]. The group untreated with peptides was a blank control. PMB and CIP were used as the positive control. Similarly, bacterial cells (1 × 10^5^ CFU/mL) were incubated with 3/128, 1/32, 3/64, 1/16, 3/32, 1/8, 3/16, 1/4, 3/8, 1/2, 3/4, 1, 2, 3, and 4 × MIC N6 or N6-COOH-miniPEG. The sample was taken from each flask at 24 h and serially diluted for colony counting [64].

### Toxicity of N6 and N6-COOH-miniPEG

Erythrocytes from SPF mice were centrifuged at 1,500 rpm for 5 min and washed with 0.9% NaCl three times. 75 μL of 8% (v/v) erythrocyte solution was mixed with 75 μL of N6 or N6-COOH-miniPEG (the final concentrations of 0.5 – 128 μg/mL); the mixture was added into 96-well plates, incubated at 37 °C for 1 h, and centrifuged at 1,500 rpm for 5 min. The supernatants were measured at 540 nm. 0.9% NaCl and 0.1% Triton X-100 were used as the blank (A_0_) and positive (A_100_) control, respectively [14, 65]. The hemolysis percentages of peptides were calculated by the following equation: Hemolysis (%) = [(A − A_0_) / (A_100_ − A_0_)] × 100.

The cytotoxicity of N6 and N6-COOH-miniPEG was evaluated using the 3-(4, 5-dimethylthiazolyl-2)- 2, 5-diphenyltetrazolium bromide (MTT) method [66]. RAW 264.7 cells (2.5 × 10^5^ cells/mL) were seeded into a 96-well plate (100 μL/well) and cultured at 37 °C (5% CO_2_, 95% saturated humidity) for 24 h. The medium was then removed and the cells were washed twice with PBS. The peptide solutions (with the concentrations from 1 to 256 μg/mL) were then added to the plates (100 μL/well), incubated for 24 h, and washed twice with PBS. Next, 5 mg/mL MTT (100 μL/well) was added and incubated for 4 h, followed by an addition of dimethyl sulfoxide (DMSO) (150 μL/well). After shaking in a shaker, the crystals in the bottom of wells were completely dissolved, and the absorbance of each well was measured at 570 nm (A_peptide_). PBS served as the control (A_control_). The cell survival rate was calculated by the equation: Survival rate (%) = (A_peptide_ / A_control_) × 100.

### Stability of N6 and N6-COOH-miniPEG

The thermal stability of peptides was determined after treatment for 1 h at different temperatures (4, 20, 40, 60, 80, and 100 °C). The antibacterial activity of peptides against *E. coli* CVCC195 was determined by the MIC assay [67]. To evaluate pH stability, the peptides were dissolved in glycine–HCl buffer (pH 2.0), sodium acetate buffer (pH 4.0), sodium phosphate buffer (pH 6.0), tris–HCl buffer (pH 8.0), or glycine–NaOH buffer (pH 10.0) and incubated for 3 h. The MIC values of the peptides against *E. coli* CVCC195 were measured as described above [40]. Similarly, salt stability of peptides was determined after a 3 h-incubation at different concentrations of NaCl solution (50, 100, 200, 300, 400, and 500 mM). Additionally, to evaluate the protease stability, N6 and N6-COOH-miniPEG (200 μg/mL) were incubated with trypsin or papain (250 U/mg, pH 8.0) for different times, an aliquot of 20 μL N6 or N6-COOH-miniPEG mixture was taken and tested against *E. coli* CVCC195 by the inhibition zone assay [68, 69]. The untreated peptides were used as the positive control and buffers alone were used as the negative control. All assays were conducted in triplicate.

Stability of N6 or N6-COOH-miniPEG in SGF and SIF was carried out as previously described [68, 70]. Briefly, N6 or N6-COOH-miniPEG (200 μg/mL) was dissolved in SGF or SIF incubated for 5 – 60 min at 37 °C. At different time intervals, an aliquot of 20 μL N6 or N6-COOH-miniPEG was taken and tested against *E. coli* CVCC195 by the inhibition zone assay. N6 or N6-COOH-miniPEG (200 μg/mL) dissolved in PBS was used as a positive control; SGF and SIF were used as the negative controls. To determine the serum of the peptides, N6 or N6-COOH-miniPEG were dissolved in PBS or mice serum at 37 °C to detect their serum stability and samples were removed at different time points to determine their residual activity by the inhibition zone or reverse-phase high-performance liquid chromatography (RP-HPLC) methods [41]. All assays were conducted in triplicate. PC-N6, equivalent N6 prepared in PBS was used as positive control; PC-N6-COOH-miniPEG, equivalent N6-COOH-miniPEG prepared in PBS was used as positive control.

### CD analysis of N6 and N6-COOH-miniPEG

The secondary structure of the peptides was determined by CD spectroscopy in ddH_2_O, SDS, and TFE, respectively [22, 69]. N6 and N6-COOH-miniPEG were dissolved in ddH_2_O, 20 mM SDS or 50% TFE and their CD spectra were measured via a MOS-450 spectropolarimeter (Bio-Logic, Grenoble, France) using a 1.0 mm path-length cuvette. The spectra of peptides were recorded from 180 to 260 nm at 25 °C at a scanning speed of 100 nm/min with a step resolution of 2.0 nm and an integration time of 2 s. Data were analyzed using CDNN software.

### Effects of N6 and N6-COOH-miniPEG on the bacterial cell membrane and membrane potential

The outer membrane permeabilization abilities of peptides were determined using the fluorescent NPN assay. Mid-log phase *E. coli* CVCC195 and *S. pullorum* CVCC533 cells were collected by centrifugation, washed twice, and suspended in n-2-hydroxyethylpiperazine-n-2-ethane sulfonic acid (HEPES) buffer (pH 7.4) to an OD_600nm_ of 0.4. Cell suspension and NPN solutions (10 μM) were added into 96-well black plates, followed by an addition of peptide solutions (1 × , 2 × , and 4 × MIC). Fluorescence intensity was recorded until no further increase with a microplate reader (excitation/emission, 328/438 nm). The cells treated with PBS were used as the negative control; the untreated cells were used as the blank control [71].

Mid-log phase *E. coli* CVCC195 and *S. pullorum* CVCC533 cells (1 × 10^8^ CFU/mL) were incubated with 1 × , 2 × and 4 × MIC peptide solutions at 37 °C for 5, 30, and 120 min, respectively. After incubation, the cells were washed three times with 0.01 M PBS, stained with PI, and analyzed using a flow cytometer (FACS Calibur, BD, USA). The data were analyzed using CellQuest Pro software (BD, USA) [72].

The DiSC_3_(5) assay was used to investigate cytoplasmic membrane depolarization [36, 73]. Mid-log phase *E. coli* CVCC195 and *S. pullorum* CVCC533 cells were washed in 5 mM HEPES buffer (pH 7.2) containing 20 mM glucose and resuspended in buffer (5 mM HEPES buffer, 20 mM glucose, and 100 mM KCl, pH 7.2) to an OD_600_ of 0.1. The fluorescence intensity of bacterial cells was monitored for 13 min. N6 and N6-COOH-miniPEG were added and the fluorescence intensity (excitation/emission, 620/670 nm) was measured using a Tecan Infinite M200 PRO microplate reader. PBS was used as the blank control. The experiment was repeated three times.

### Interaction between N6 or N6-COOH-miniPEG and bacterial LPS

The affinity between the peptides and LPS was detected by the fluorescent probe BC [74]. Equal volumes of LPS (40 μg/mL) and BC probe (10 μM) in 50 mM Tris buffer (pH 7.4) were mixed and added into a black 96-well microtiter plate (180 μL/well), followed by an addition of different concentrations of N6 or N6-COOH-miniPEG (20 μL/well). Fluorescence was measured by a Tecan Infinite M200 PRO microplate reader at room temperature (excitation/emission, 580/620 nm) and the BC displacement rate was calculated as previously described [41].

### Effects of N6 and N6-COOH-miniPEG on the bacterial morphologies

Mid-log phase *E. coli* CVCC195 and *S. pullorum* CVCC533 cells (1 × 10^8^ CFU/mL) were incubated with 4 × MIC N6 or N6-COOH-miniPEG for 2 h at 37 °C. After incubation, the cells were washed with 0.01 M PBS for three times and fixed with 2.5% glutaric dialdehyde overnight at 4 °C. The cells were dehydrated with ethanol series (50%, 70%, 85%, 95%, and 100%, × 3 times) for 15 min/time, dried by CO_2_, sputtered with platinum, and observed using a QUANTA200 SEM (FEI, Philips, Netherlands) [75].

For TEM observations, the bacterial cells were treated with peptides and fixed as described above. After fixation, bacterial cells were washed with 0.01 M PBS for five times and post-fixed in 1% osmium tetroxide (OsO_4_) for 1 h. The cells were dehydrated with a series of graded ethanol solutions (50%, 70%, 85%, 95%, and 100%, × 2 times) for 15 min/time, transferred to epoxy resins, and stained with 1% uranium acetate; the cells were observed using a JEM1400 TEM (JEDL, Japan) [14].

### Effects of N6 and N6-COOH-miniPEG on cytokines in macrophages challenged with LPS

RAW 264.7 cells (2.5 × 10^5^ cells/mL) were cultured in 12-well plates. N6 or N6-COOH-miniPEG (100 μg/mL) was mixed with LPS (100 ng/mL) for 30 min and added into the cells. After treatment for 4 h at 37 °C, the supernatant from each well was collected, and the levels of TNF-α, IL-6, IL-1β, and IL-10 were determined by the ELISA [76]. The cells challenged with LPS (100 ng/mL) were used as the negative control (LPS), and the cells without challenge and treatment were used as the blank control (CK).

### Biodistribution of N6 and N6-COOH-miniPEG in mice

The 6-week-old female nude mice (weight 20 ± 2 g) were intraperitoneally injected with 10 mg/kg FITC-labeled N6 and N6-COOH-miniPEG, respectively. Free FITC was used as the negative control. Real-time fluorescence in mice was observed at 0.17, 0.5, 0.75, 1, 1.5, 2, 3, 4, 6, and 24 h, respectively, with the excitation wavelength at 488 nm using a Maestro 2 IVIS® Spectrum CT (PerkinElmer, USA) [41, 77].

### Efficacy of N6 and N6-COOH-miniPEG in a mouse peritonitis model

To evaluate in vivo efficacy of N6 and N6-COOH-miniPEG, the 6-week-old female ICR mice, weight 20 ± 2 g (10 mice/group) were intraperitoneally injected with *E. coli* CVCC195 or *S. pullorum* CVCC533 at a concentration of absolute lethal dose (LD100) [78]. After the intraperitoneal injection with *E. coli* CVCC195 (1 × 10^9^ CFU/mL, 0.2 mL), the mice were treated with N6 (2 and 4 μmol/kg), N6-COOH-miniPEG (2 and 4 μmol/kg) or PMB (0.125 and 0.25 μmol/kg) at 0.5 h and 8 h, respectively.

Similarly, after the intraperitoneal injection with *S. pullorum* CVCC533 (5 × 10^7^ CFU/mL, 0.2 mL), the mice were treated with N6 (8 and 10 μmol/kg), N6-COOH-miniPEG (8 and 10 μmol/kg) or CIP (0.8 and 1 μmol/kg) at 0.5 h, respectively. The mice injected with antibiotics or PBS served as the positive and blank control (CK), respectively. Survival of the mice was recorded daily for 7 d.

The mice (10 mice/group) were challenged with *E. coli* CVCC195 (1 × 10^9^ CFU/mL, 0.2 mL) or *S. pullorum* CVCC533 (5 × 10^7^ CFU/mL, 0.2 mL) by intraperitoneal injection and treated with N6 (4 and 10 μmol/kg) or N6-COOH-miniPEG (4 and 10 μmol/kg). To evaluate bacterial loads in organs, the livers, spleens, kidneys and lungs were removed from the sacrificed mice at 24 h post treatment and homogenized in sterile PBS for *E. coli* CVCC195 colony counting; similarly, the livers, spleens, kidneys and lungs were removed from the sacrificed mice at 48 h after treatment and homogenized in sterile PBS for *S. pullorum* CVCC533 colony counting. The uninfected mice served as the blank control (CK); the infected mice treated with PBS were used as the negative control. The mice were sacrificed by neck dislocation under inhaled isoflurane anesthesia (RWD Life Science Co., LTD. Shenzhen, China).

The mice (10 mice/group) were injected intraperitoneally with N6 and N6-COOH-miniPEG (0.25 μmol/kg), PMB (10 μmol/kg) or CIP (5 μmol/kg) 0.5 h after challenge with 10 mg/kg *E. coli* 0111:B4 LPS. Blood was collected in a heparin sodium tube at 2 or 8 h after LPS injection and incubated for 30 min at 37 °C and then overnight at 4 °C. After centrifugation at 3,000 rpm for 10 min at 4 °C, serum was collected from supernatant. The cytokine levels of TNF-α, IL-6, IL-1β and IL-10 in serum were detected by the Jiaxuan Biotech. Co. Ltd. (Beijing, China) using an ELISA. RAW 264.7 cells untreated with LPS and antimicrobial drugs were used as a blank control (CK).

The mice were treated with N6 (4 and 10 μmol/kg) and N6-COOH-miniPEG (4 and 10 μmol/kg) after intraperitoneal injection of *E. coli* CVCC195 for 0.5 and 8 h or *S. pullorum* CVCC533 for 0.5 h as described above. Livers, spleens, kidneys and lungs were removed from the mice at 24 h and 5 d post-treatment after *E. coli* CVCC195 injection and at 48 h and 4 d post-treatment after *S. pullorum* CVCC533 injection, respectively, washed in PBS, fixed in 4% paraformaldehyde at 4 °C for 24 h, and stained with hematoxylin and eosin (HE) for histopathology analysis. Finally, the tissue samples were fixed and observed by the light microscope. The uninfected mice served as the blank control (CK); the infected mice being treated with PBS were used as the negative control.

## Statistics

All data were analyzed by Origin 2018pro or GraphPad Prism 7. The results were given as means ± standard error (SE). Statistical analyses between treatments or groups were determined using one-way analysis of variance (ANOVA) models in SAS 9.2 (SAS Institute Inc., Cary, NC, USA), followed by Dunnett's multiple comparisons test. A *p*-value of < 0.05 was considered statistically significant.

## Supplementary Information

**Additional file 1:** **Figure S1.** Chemicalstructure of N6. **Figure S2.** Chemicalstructure of N6-COOH-miniPEG. **Figure S3. **Chemical structure of N6-NH_2_-miniPEG.**Figure S4. **Chemical structure of N6-NH_2_-PEG6. **Figure S5.** Chemicalstructure of N6-NH_2_-PEG12. **Figure S6. **Chemical structure of N6-NH_2_-PEG24. **Figure S7.** Chemicalstructure of N6-Cys7-miniPEG. **Figure S8.** Chemicalstructure of N6-Cys16-miniPEG. **Figure S9. **CD of N6 and N6-COOH-miniPEG indifferent solutions. **Figure S10.** Interaction of N6 and N6-COOH-miniPEG with cell membrane. Outer membrane permeabilization of *E. coli *CVCC195(**A**) and *S. pullorum* CVCC533 (**B**) cells after treated with N6and N6-COOH-miniPEG. **Figure S11.** Interactionof N6 and N6-COOH-miniPEGwith cell membrane. Inner membranepermeabilization of *E. coli *CVCC195 (**A**) and *S. pullorum* CVCC533 (**B**) cells. Bacterial cells were treated with 1×, 2× or 4× MIC for 5, 30 or 120 minand analyzed by flow cytometry. **Figure S12. **Interactionof N6 and N6-COOH-miniPEGwith cell membrane. (**A-D**) Effects of N6 and N6-COOH-miniPEG on *E. coli *CVCC195 (**A,B**) and *S. pullorum *CVCC533 (**C, D**) cytoplasmicmembrane potential. **Figure S13.** Binding affinity of N6 andN6-COOH-miniPEG to LPS. **Figure S14. **TEM images of *E. coli *CVCC195 and *S. pullorum*CVCC533 cells treated with N6 and N6-COOH-miniPEG. After treatment with 4 × MIC N6 or N6-COOH-miniPEGfor 2 h, *E. coli *CVCC195 and *S. pullorum* CVCC533 cells weredehydrated, sputtered, and observed on JEM1400 (JEDL, Tokyo, Japan). **Figure S15. **Effects of N6 and its N6-COOH-miniPEG on organ injury in mice.The mice were infectedintraperitoneally with* E. coli *CVCC195 (1×10^9^ CFU/mL, 200 μL)and treated with N6 (4 μmol/kg) or N6-COOH-PEG (4 μmol/kg). The livers,spleens, kidneys and lungs were harvested from the mice sacrificed at 5 d afterinfection. **Figure S16.** Effectsof N6 and its N6-COOH-miniPEG on organ injury in mice. The mice wereinfected intraperitoneally with* S. pullorum *CVCC533 (5 × 10^7^CFU/mL, 200 μL) and treated with N6 (10 μmol/kg) or N6-COOH-PEG (10 μmol/kg).The livers, spleens, kidneys and lungs were harvested from the mice sacrificedat 48 h after infection. **Figure S17. **Effects ofN6 and N6-COOH-miniPEGon organ injury in mice.Mice were infectedintraperitoneally with* S. pullorum *CVCC533 (5 × 10^7^ CFU/mL,200 μL) and treated with N6 (10 μmol/kg) or N6-COOH-miniPEG (10 μmol/kg). Livers, spleens, kidneys and lungs were harvested from mice sacrificed at 4 dafter infection. CK group: The livers, spleens, kidneys, and lungs were normal; Infected *S. pullorum *CVCC533 group: Thereis “bridgingnecrosis” around the confluent area of the liver, a marked decrease in the density oflymphocytes in the splenic nodes, diffuse inflammatory cell infiltration in therenal interstitium, “honeycomb” changes throughout the lung tissue, and a large amount of bloody exudate in thealveolar cavity (arrow) (× 200,scale bar = 100 μm); N6 treatment group: Foci of hepatocyte necrosis are seenin the lobules of the liver, localised reduced lymphocyte density in thesplenic nodules, localised renal tubular degeneration and atrophy, and a smallamount of inflammatory cell infiltration in the interstitial lung (arrow) (× 200, scale bar = 100 μm); N6-COOH-miniPEG treatment group: Focal foci of hepatocyte necrosis are seen inthe lobules of the liver, localised reduced lymphocyte density in the splenicnodules, localised renal tubular degeneration and atrophy, and a small amountof scattered inflammatory cell infiltration in the interstitial lung (× 200, scale bar = 100 μm); CIP treatment group: Localized inflammatory cell infiltration in the hepaticsinusoids, reduced lymphocyte density in the splenic nodules, localizedscattered inflammatory cell infiltration in the renal interstitium, markedwidening of the alveolar septa and narrowing of the alveolar cavity (× 200, scale bar = 100 μm). **Table S1. **Proportion of secondary structure of N6 and N6-COOH-miniPEG in different solutions. **Table S2. **MIC values (μg/mL) of N6 and N6-COOH-miniPEG against *E. coli *CVCC195 in differentconditions.

**Additional file 2.** (XLSX 89 kb)

## Data Availability

All data generated or analysed during this study are included in this published article (and its supplementary information files).

## Abbreviations

PEG: Polyethylene glycol
TNF: Tumor necrosis factor
IL: Interleukin
AMPs: Antimicrobial peptides
MWs: Molecular weights
MIC: Minimum inhibitory concentration
SGF: Simulated gastric fluid
SIF: Simulated intestinal fluid
CD: Circular dichroism
SDS: Sodium dodecyl sulfate
TFE: Trifluoroethanol
NPN: N-phenyl-1-naphthylamine
PI: Propidium iodide
DiSC_3_(5): 3,3′-Dipropylthiadicarbocyanine iodide
LPS: Lipopolysaccharide
BC: BODIPY-TR cadaverine
SEM: Scanning electron microscopy
ELISA: Enzyme-linked immunosorbent assay
FITC: Fluorescein isothiocyanate
PMB: Polymyxin B
FDA: Food and drug administration
GIP: Glucose-dependent insulinotropic polypeptide
TEM: Transmission electron microscopy
SPF: Specific-pathogen-free
ESI–MS: Electrospray ionization mass spectrometry
CLSI: Clinical and Laboratory Standards Institute
MTT: 3-(4, 5-Dimethylthiazolyl-2)- 2, 5-diphenyltetrazolium bromide
DMSO: Dimethyl sulfoxide
RP-HPLC: Reversed phase high performance liquid chromatography
HEPES: N-2-hydroxyethylpiperazine-n-2-ethane sulfonic acid
OSO_4_: Osmium tetroxide
HE: Hematoxylin and eosin
SE: Standard error
