# Supplementary material for: C-terminal mini-PEGylation of a marine peptide N6 had potent antibacterial and anti-inflammatory properties against Escherichia coli and Salmonella strains in vitro and in vivo
Source: BMC Microbiol. 2022 May 12;22:128. doi: 10.1186/s12866-022-02534-w (PMC9097129; doi:10.1186/s12866-022-02534-w)
Supplement: Supplementary file 1 — Additional file 1: Figure S1. Chemicalstructure of N6. Figure S2. Chemicalstructure of N6-COOH-miniPEG. Figure S3. Chemical structure of N6-NH2-miniPEG.Figure S4. Chemical structure of N6-NH2-PEG6. Figure S5. Chemicalstructure of N6-NH2-PEG12. Figure S6. Chemical structure of N6-NH2-PEG24. Figure S7. Chemicalstructure of N6-Cys7-miniPEG. Figure S8. Chemicalstructure of N6-Cys16-miniPEG. Figure S9. CD of N6 and N6-COOH-miniPEG indifferent solutions. Figure S10. Interaction of N6 and N6-COOH-miniPEG with cell membrane. Outer membrane permeabilization of E. coli CVCC195(A) and S. pullorum CVCC533 (B) cells after treated with N6and N6-COOH-miniPEG. Figure S11. Interactionof N6 and N6-COOH-miniPEGwith cell membrane. Inner membranepermeabilization of E. coli CVCC195 (A) and S. pullorum CVCC533 (B) cells. Bacterial cells were treated with 1×, 2× or 4× MIC for 5, 30 or 120 minand analyzed by flow cytometry. Figure S12. Interactionof N6 and N6-COOH-miniPEGwith cell membrane. (A-D) Effects of N6 and N6-COOH-miniPEG on E. coli CVCC195 (A,B) and S. pullorum CVCC533 (C, D) cytoplasmicmembrane potential. Figure S13. Binding affinity of N6 andN6-COOH-miniPEG to LPS. Figure S14. TEM images of E. coli CVCC195 and S. pullorumCVCC533 cells treated with N6 and N6-COOH-miniPEG. After treatment with 4 × MIC N6 or N6-COOH-miniPEGfor 2 h, E. coli CVCC195 and S. pullorum CVCC533 cells weredehydrated, sputtered, and observed on JEM1400 (JEDL, Tokyo, Japan). Figure S15. Effects of N6 and its N6-COOH-miniPEG on organ injury in mice.The mice were infectedintraperitoneally with E. coli CVCC195 (1×10^9^ CFU/mL, 200 μL)and treated with N6 (4 μmol/kg) or N6-COOH-PEG (4 μmol/kg). The livers,spleens, kidneys and lungs were harvested from the mice sacrificed at 5 d afterinfection. Figure S16. Effectsof N6 and its N6-COOH-miniPEG on organ injury in mice. The mice wereinfected intraperitoneally with S. pullorum CVCC533 (5 × 10^7^CFU/mL, 200 μL) and treated with N6 (10 μmol/kg) or N6-COOH-PEG (10 μmol/kg).Th [file 12866_2022_2534_MOESM1_ESM.docx]

**Supplementary Material**

**C-terminal mini-PEGylation of** **a marine peptide N6 had potent antibacterial** **and anti-inflammatory properties against *Escherichia coli* and *Salmonella* strains *in vitro* and *in vivo***

Ting Li^1,^^2^, Na Yang^1,^^2^, Da Teng^1,2^, Ruoyu Mao^1,2^, Ya Hao^1,2^, Xiumin Wang^1,2,3*^ and Jianhua Wang^1,2*^

^1^Gene Engineering Laboratory, Feed Research Institute, Chinese Academy of Agricultural Sciences, Beijing 100081, People’s Republic of China

^2^Key Laboratory of Feed Biotechnology, Ministry of Agriculture and Rural Affairs, Beijing 100081, People’s Republic of China

^3^Chinese Herbal Medicine Laboratory, Feed Research Institute, Chinese Academy of Agricultural Sciences, Beijing 100081, People’s Republic of China

*Corresponding author

Prof., Ph.D., PI. Jianhua Wang and postal address of all authors as:

Gene Engineering Laboratory, Feed Research Institute

Chinese Academy of Agricultural Sciences, 12 Zhongguancun Nandajie St., Haidian District, Beijing 100081, People’s Republic of China

E-mail address: wangxiumin@caas.cn; wangjianhua@caas.cn

Phone: 0086-10-82106081, 0086-10-82106079; Fax: 0086-10-82106079


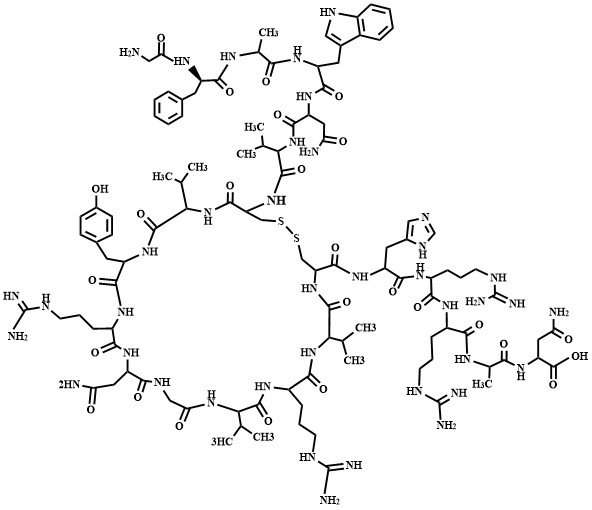


**Figure S1.** **Chemical structure of N6.**


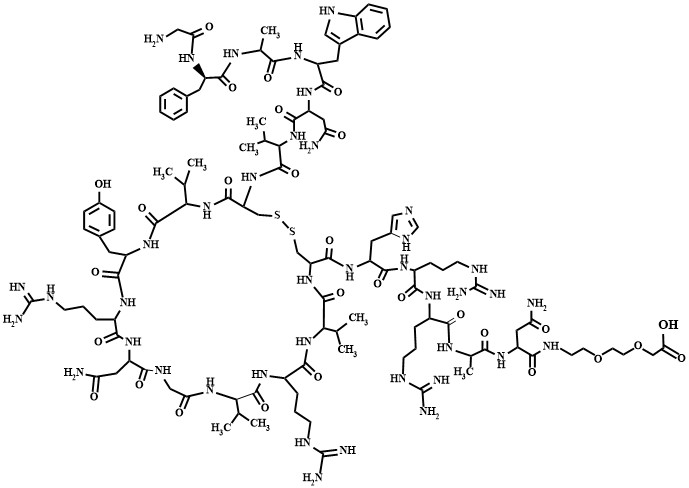


**Figure S2.** **Chemical structure of N6-COOH-miniPEG.**


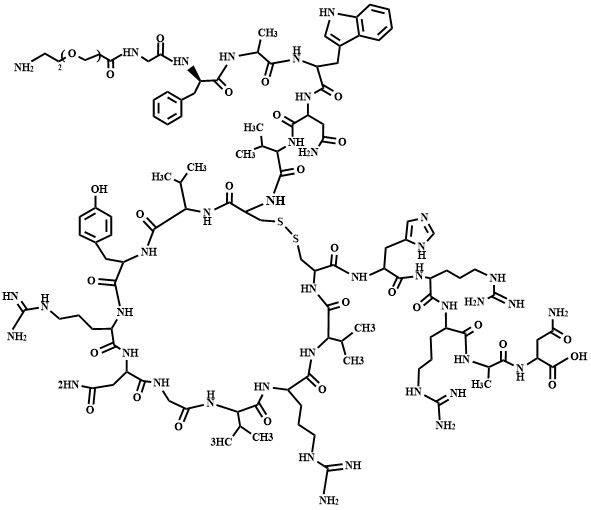


**Figure S3. Chemical structure of N6-NH_2_-miniPEG.**


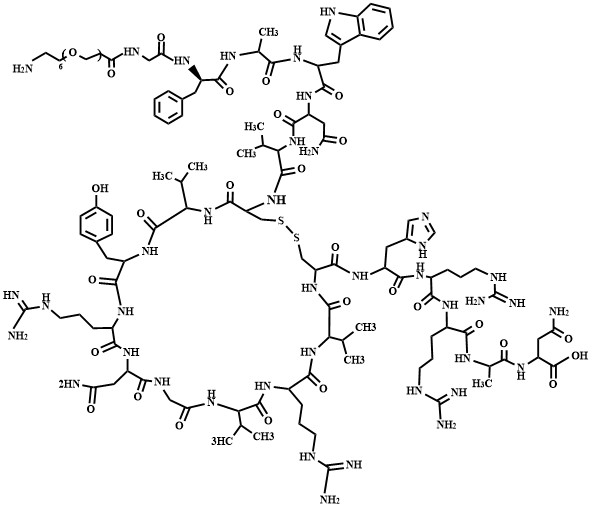


**Figure S4. Chemical structure of N6-NH_2_-PEG6.**


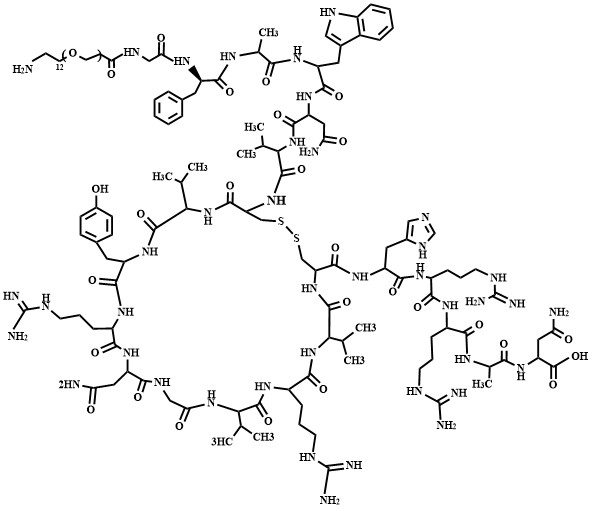


**Figure S5.** **Chemical structure of N6-NH_2_-PEG12.**


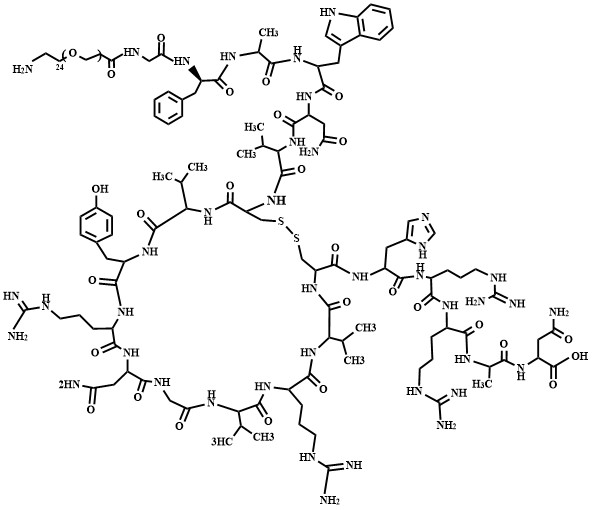


**Figure S6. Chemical structure of N6-NH_2_-PEG24.**


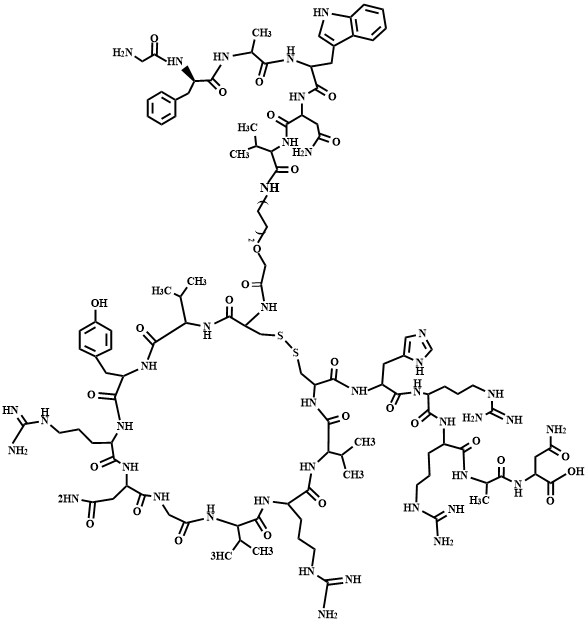


**Figure S7.** **Chemical structure of N6-Cys7-miniPEG.**


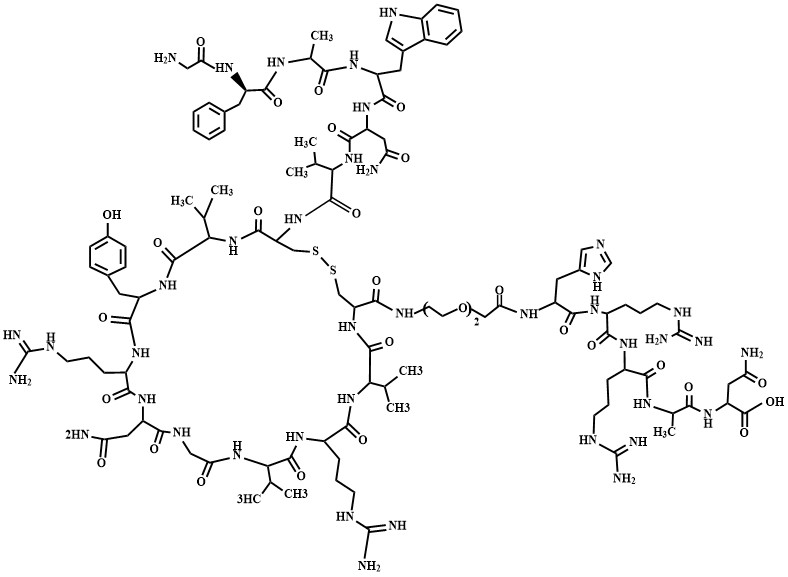


**Figure S8.** **Chemical structure of N6-Cys16-miniPEG.**

**

**

**Figure S9. CD of N6 and N6-COOH-miniPEG in different solutions.**

**
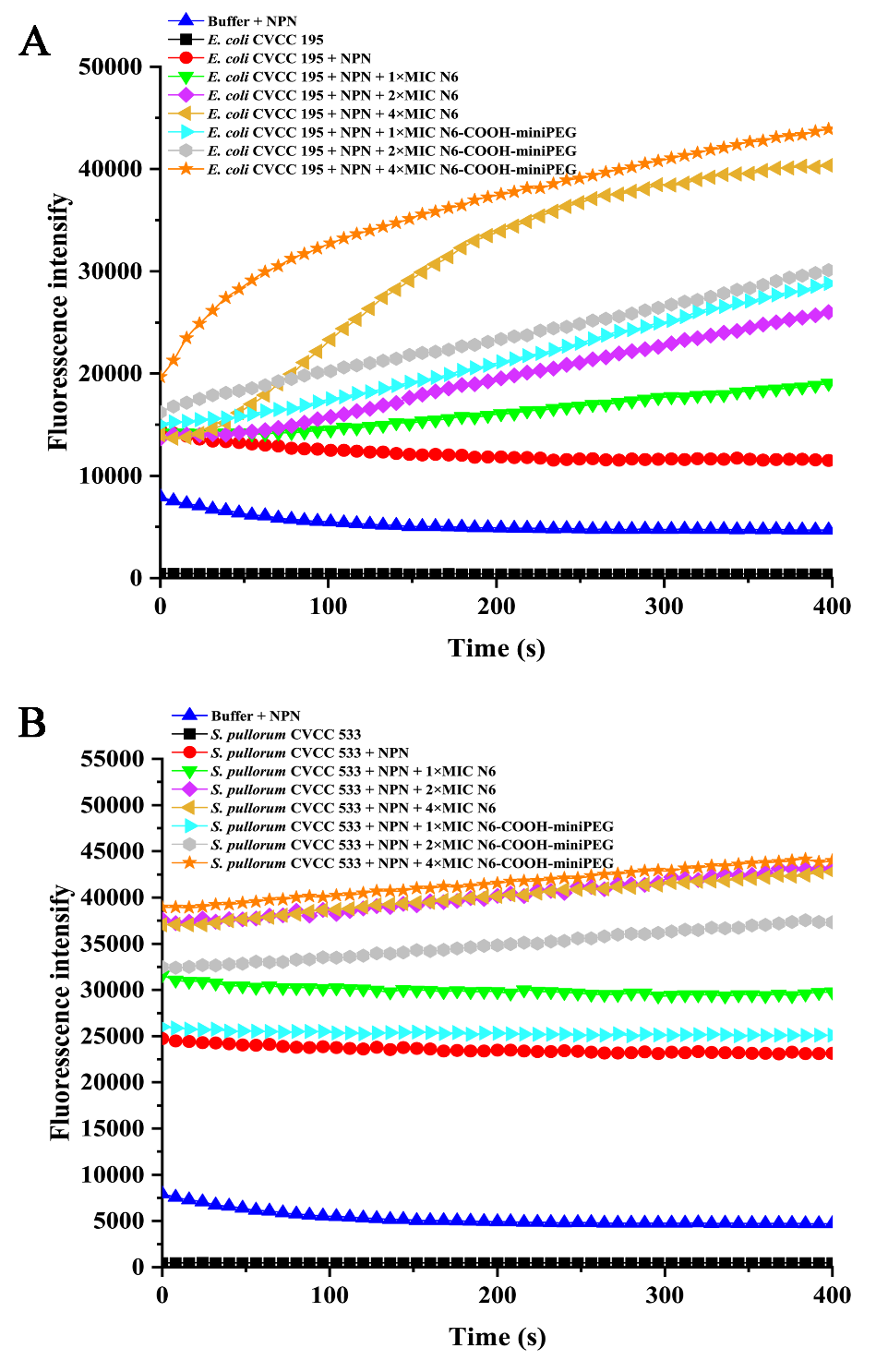
**

**Figure S10.** **Interaction of N6 and N6-COOH-miniPEG with cell membrane.** Outer membrane permeabilization of *E. coli* CVCC195 (**A**) and *S. pullorum* CVCC533 (**B**) cells after treated with N6 and N6-COOH-miniPEG.


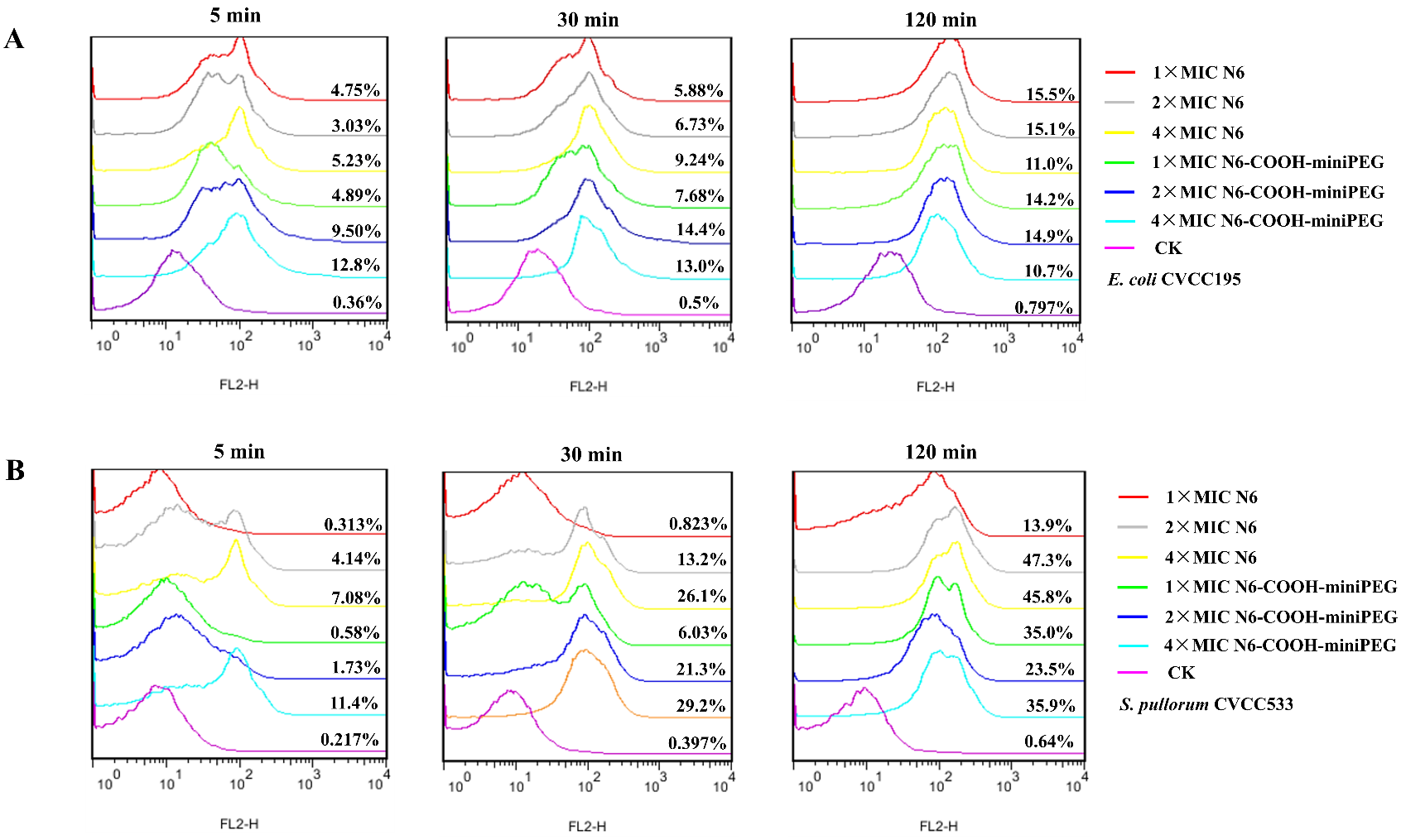


**Figure S11.** **Interaction of N6 and N6-COOH-miniPEG with cell membrane.** Inner membrane permeabilization of *E. coli* CVCC195 (**A**) and *S. pullorum* CVCC533 (**B**) cells. Bacterial cells were treated with 1×, 2× or 4× MIC for 5, 30 or 120 min and analyzed by flow cytometry.

**
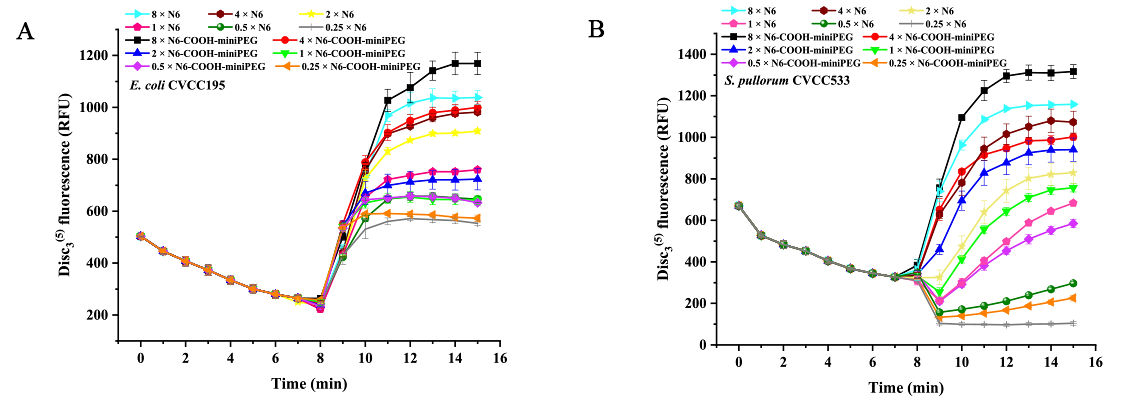
**

**Figure S12.** Interaction of N6 and N6-COOH-miniPEG with cell membrane. (**A-D**) Effects of N6 and N6-COOH-miniPEG on *E. coli* CVCC195 (**A, B**) and *S. pullorum* CVCC533 (**C, D**) cytoplasmic membrane potential.


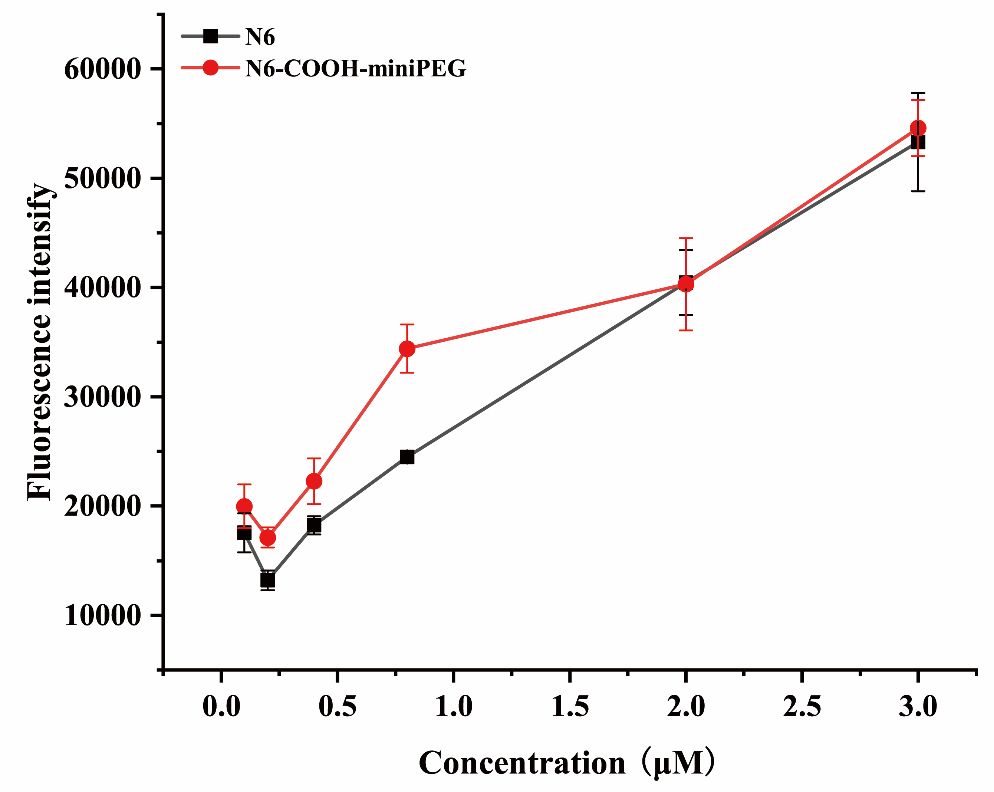


**Figure S13.** **Binding affinity of N6 and N6-COOH-miniPEG to LPS.**


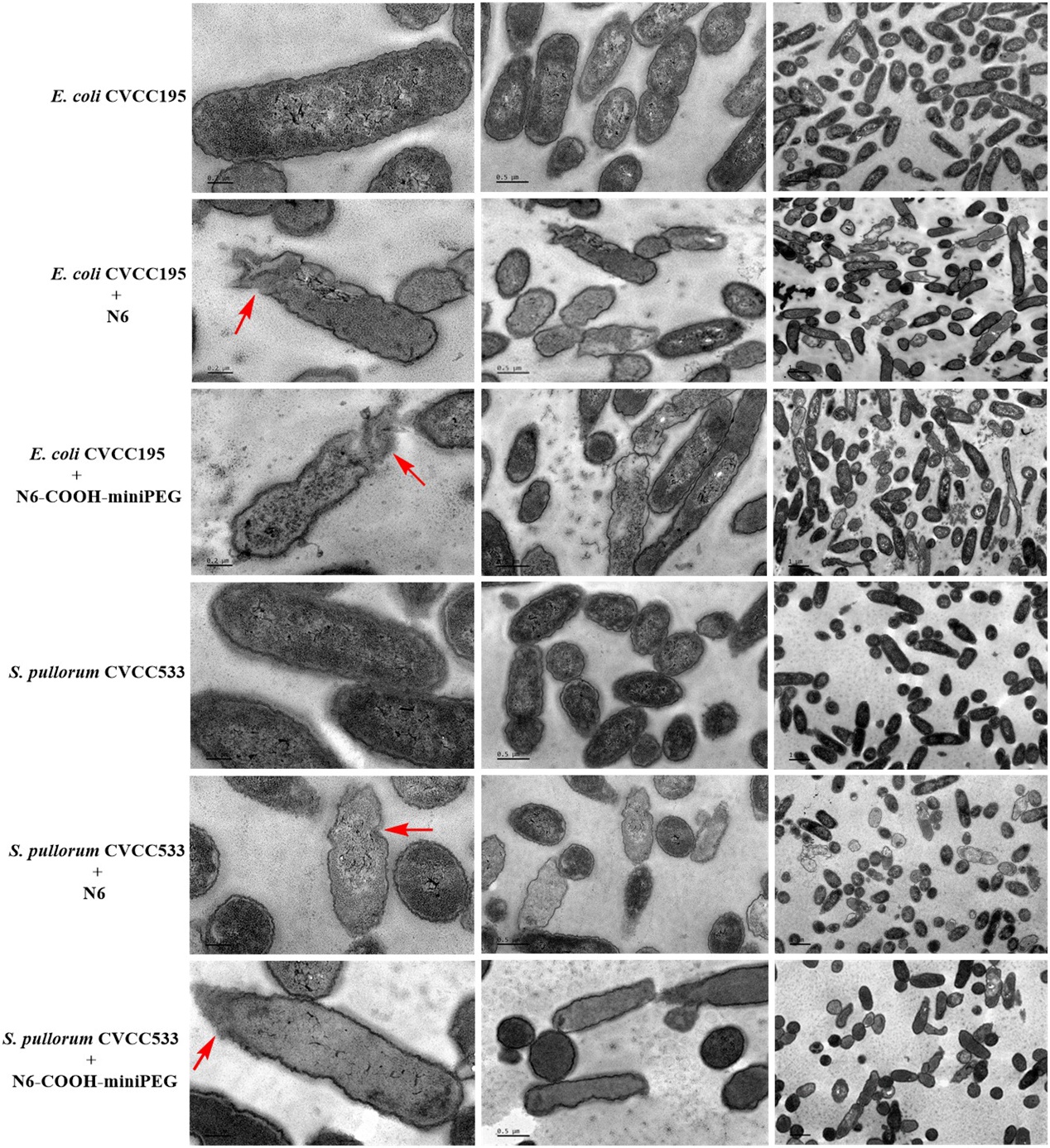


**Figure S14. TEM images of *E. coli* CVCC195 and *S. pullorum* CVCC533 cells treated with N6 and N6-COOH-miniPEG.** After treatment with 4 × MIC N6 or N6-COOH-miniPEG for 2 h, *E. coli* CVCC195 and *S. pullorum* CVCC533 cells were dehydrated, sputtered, and observed on JEM1400 (JEDL, Tokyo, Japan).


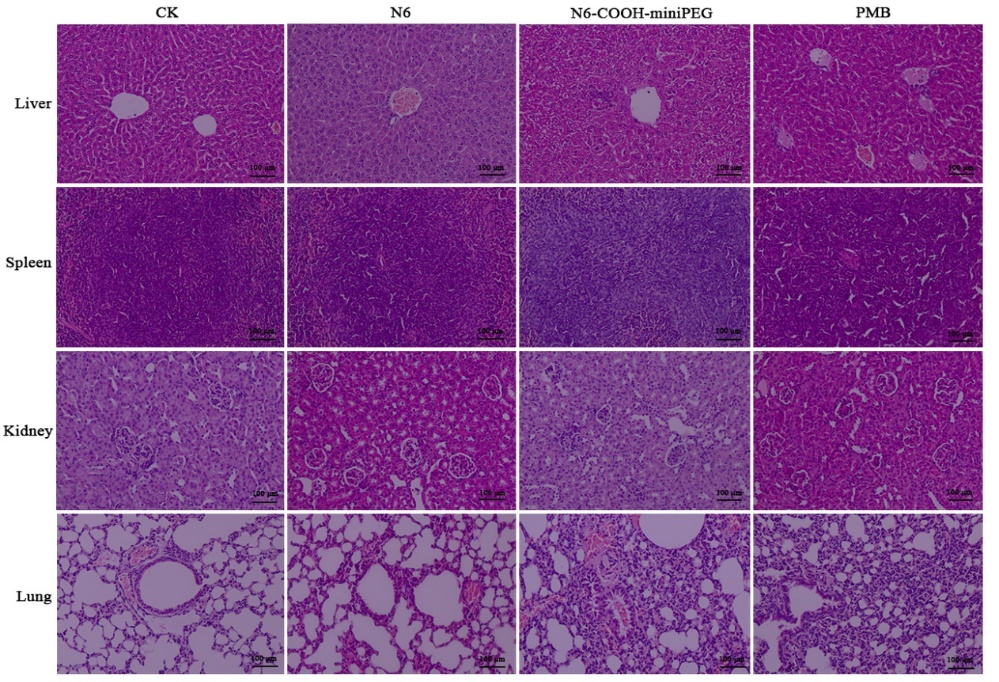


**Figure S15. Effects of N6 and its N6-COOH-miniPEG on organ injury in mice.** The mice were infected intraperitoneally with *E. coli* CVCC195 (1×10^9^ CFU/mL, 200 μL) and treated with N6 (4 μmol/kg) or N6-COOH-PEG (4 μmol/kg). The livers, spleens, kidneys and lungs were harvested from the mice sacrificed at 5 d after infection.


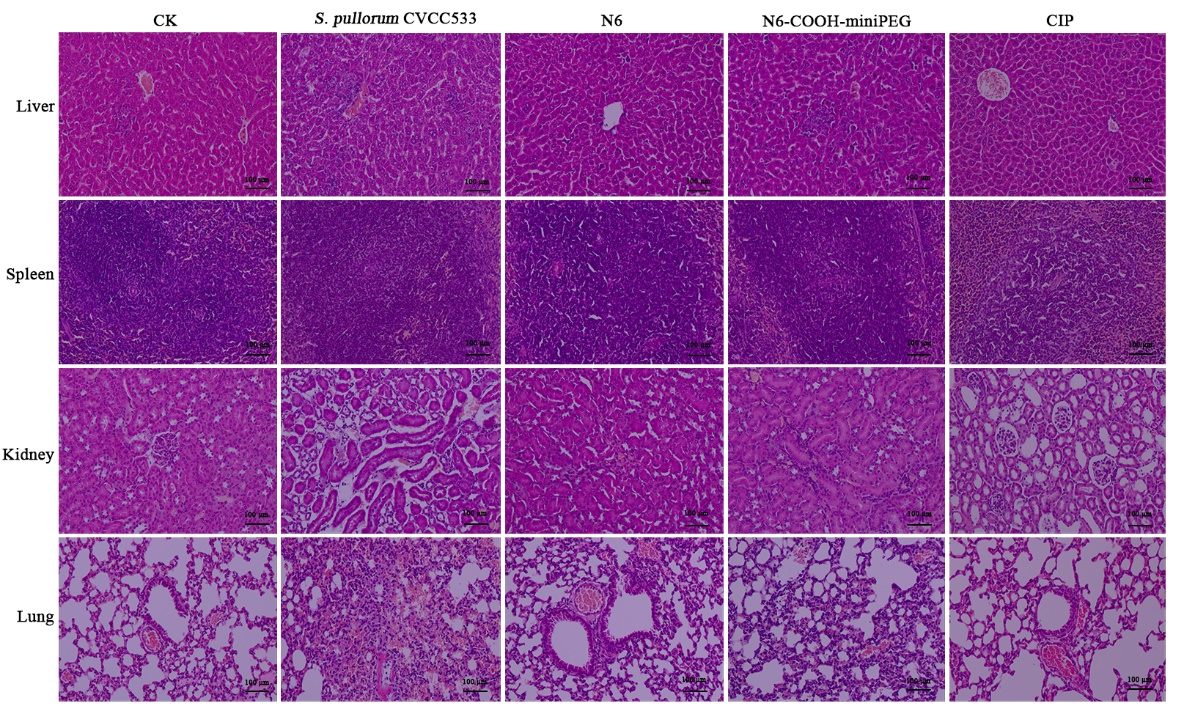


**Figure S16.** **Effects of N6 and its N6-COOH-miniPEG on organ injury in mice.** The mice were infected intraperitoneally with *S. pullorum* CVCC533 (5×10^7^ CFU/mL, 200 μL) and treated with N6 (10 μmol/kg) or N6-COOH-PEG (10 μmol/kg). The livers, spleens, kidneys and lungs were harvested from the mice sacrificed at 48 h after infection.

*
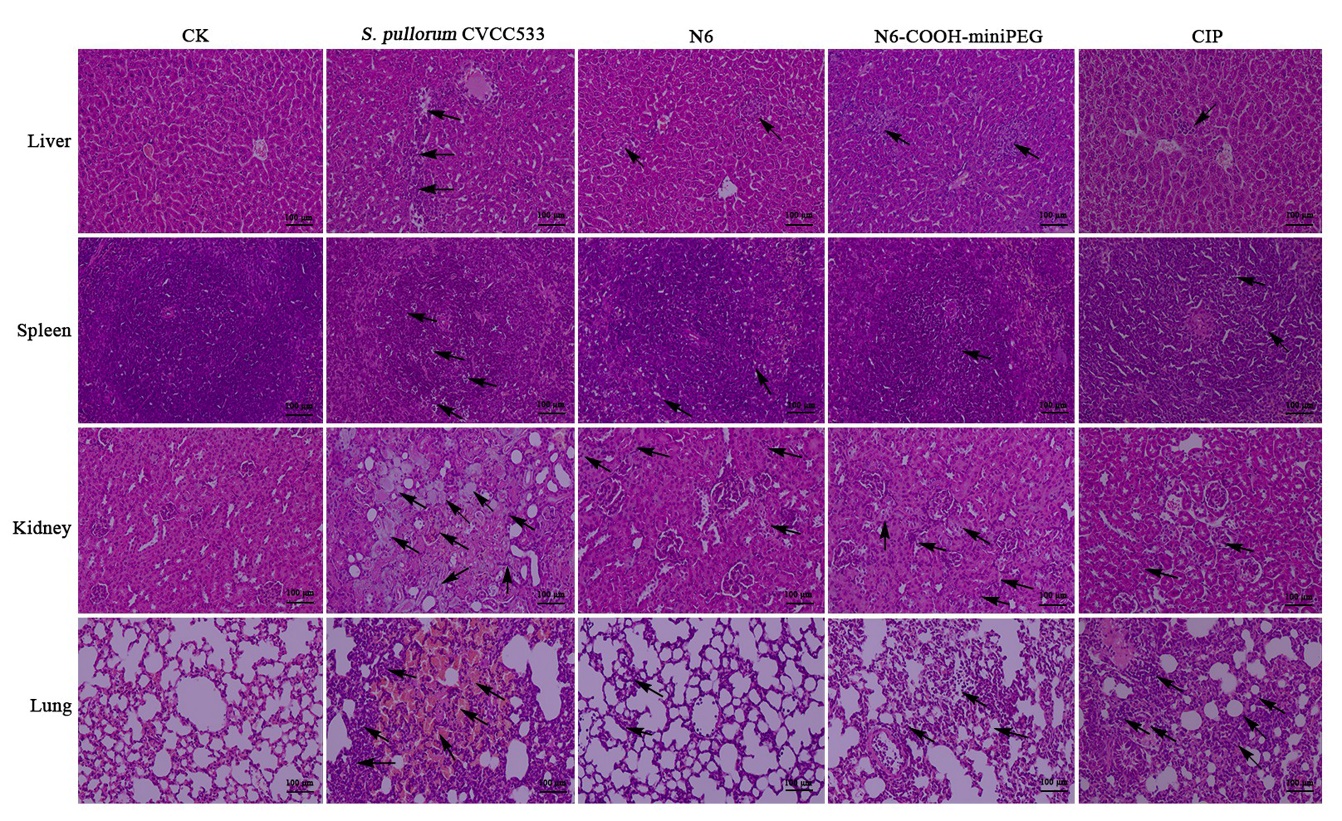
*

**Figure S17. Effects of N6 and N6-COOH-miniPEG on organ injury in mice.** Mice were infected intraperitoneally with *S. pullorum* CVCC533 (5 × 10^7^ CFU/mL, 200 μL) and treated with N6 (10 μmol/kg) or N6-COOH-miniPEG (10 μmol/kg). Livers, spleens, kidneys and lungs were harvested from mice sacrificed at 4 d after infection. CK group: The livers, spleens, kidneys, and lungs were normal; Infected *S. pullorum* CVCC533 group: There is “bridging necrosis” around the confluent area of the liver, a marked decrease in the density of lymphocytes in the splenic nodes, diffuse inflammatory cell infiltration in the renal interstitium, “honeycomb” changes throughout the lung tissue, and a large amount of bloody exudate in the alveolar cavity (arrow) (× 200, scale bar = 100 μm); N6 treatment group: Foci of hepatocyte necrosis are seen in the lobules of the liver, localised reduced lymphocyte density in the splenic nodules, localised renal tubular degeneration and atrophy, and a small amount of inflammatory cell infiltration in the interstitial lung (arrow) (× 200, scale bar = 100 μm); N6-COOH-miniPEG treatment group: Focal foci of hepatocyte necrosis are seen in the lobules of the liver, localised reduced lymphocyte density in the splenic nodules, localised renal tubular degeneration and atrophy, and a small amount of scattered inflammatory cell infiltration in the interstitial lung (× 200, scale bar = 100 μm); CIP treatment group: Localized inflammatory cell infiltration in the hepatic sinusoids, reduced lymphocyte density in the splenic nodules, localized scattered inflammatory cell infiltration in the renal interstitium, marked widening of the alveolar septa and narrowing of the alveolar cavity (× 200, scale bar = 100 μm).

**Table S1** Proportion of secondary structure of N6 and N6-COOH-miniPEG in different solutions.

|  | ddH_2_O (%) | |  | 20 mM SDS (%) | |  | 50% TFE (%) | |
| --- | --- | --- | --- | --- | --- | --- | --- | --- |
|  | N6 | N6-COOH-miniPEG |  | N6 | N6-COOH-miniPEG |  | N6 | N6-COOH-miniPEG |
| Helix | 5.9 | 5.8 |  | 8.6 | 10.4 |  | 9.8 | 12 |
| Antiparallel | 21.2 | 27.1 |  | 20.3 | 33.3 |  | 32.9 | 42.6 |
| Parallel | 2.5 | 2.7 |  | 2.9 | 3.9 |  | 3.5 | 3.4 |
| Beta-turn | 29.2 | 26.5 |  | 27.4 | 20.2 |  | 25.7 | 23.7 |
| Rndm coil | 41.2 | 37.9 |  | 40.8 | 32.1 |  | 28.1 | 18.2 |

**Table S2** MIC values (μg/mL) of N6 and N6-COOH-miniPEG against *E. coli* CVCC195 in different conditions.

| Peptide | Control | Temperatures (℃) | | | | | |  | pH values | | | | |  | Physiological salts (mM) | | | | | |  | Enzymes | | | |
| --- | --- | --- | --- | --- | --- | --- | --- | --- | --- | --- | --- | --- | --- | --- | --- | --- | --- | --- | --- | --- | --- | --- | --- | --- | --- |
|  |  | 4 | 20 | 40 | 60 | 80 | 100 |  | 2 | 4 | 6 | 8 | 10 |  | 50 | 100 | 200 | 300 | 400 | 500 |  | Pepsin | | Trypsin | Proteinase K |
| N6 | 4 | 4 | 4 | 4 | 4 | 8 | 8 |  | 2 | 8 | 8 | 4 | 4 |  | 8 | 8 | 8 | 8 | 8 | 8 |  | 8 | > 128 | | > 128 |
| N6-COOH-miniPEG | 8 | 8 | 8 | 8 | 8 | 8 | 8 |  | 4 | 16 | 8 | 8 | 8 |  | 8 | 8 | 8 | 8 | 8 | 8 |  | 8 | > 128 | | > 128 |
